# Phenolic constituents from twigs of *Aleurites fordii* and their biological activities

**DOI:** 10.3762/bjoc.17.151

**Published:** 2021-09-07

**Authors:** Kyoung Jin Park, Won Se Suh, Da Hye Yoon, Chung Sub Kim, Sun Yeou Kim, Kang Ro Lee

**Affiliations:** 1 Natural Products Laboratory, School of Pharmacy, Sungkyunkwan University, Suwon 16419, Republic of Korea; 2 Laboratory of Pharmacognosy, College of Pharmacy, Gachon University, Incheon, 21936, Republic of Korea; 3 Department of Biopharmaceutical Convergence, Sungkyunkwan University, Suwon 16419, Republic of Korea

**Keywords:** *Aleurites fordii*, antineuroinflammation, Euphorbiaceae, neolignan glycoside, neuroprotective activity, phenolic compound

## Abstract

Three new neolignan glycosides (**1**–**3**), a new phenolic glycoside (**15**), and a new cyanoglycoside (**16**) were isolated and characterized from the twigs of *Aleurites fordii* together with 14 known analogues (**4**–**14** and **17**–**19**). The structural elucidation of the new compounds was performed through the analysis of their NMR, HRMS, and ECD spectra and by chemical methods. All isolated compounds were tested for their antineuroinflammatory and neuroprotective activities.

## Introduction

*Aleurites fordii* Hemsl. (= *Vernicia fordii* Hemsl., Euphorbiaceae), known as tung oil tree, is widely distributed throughout Northeast Asia [1]. The fruits, leaves, and roots of this plant have been used as a Korean traditional medicine for treating sore throat, respiratory illness, constipation, and dieresis [2–3]. Phytochemical investigations of *A. fordii* reported coumarins, diterpenoid esters, triterpenoids, and tannins [4–7]. Some phorbol diterpenoids isolated from *A. fordii* have shown Epstein–Barr virus activation effects and an enhancement of HTLV-I-induced colony formation of lymphocytes [8].

As an ongoing search for bioactive secondary metabolites from Korean medicinal sources, we investigated the methanolic extract of the twigs of *A. fordii* which resulted in the isolation and characterization of 14 lignan derivatives including three new neolignan glycosides (**1**–**3**), four phenolic glycosides including a new compound (**15**), and a new cyanoglycoside (**16**) from the organic extracts. The structures of the new compounds were established by NMR analysis (^1^H and ^13^C NMR, COSY, HSQC, HMBC, and NOESY), HRMS, and chemical methods. The isolated compounds **1**–**19** were evaluated for their antineuroinflammatory and neuroprotective activities. In this paper, we report the isolation and structural elucidation of these phytochemicals and their biological activity.

## Results and Discussion

**Figure 1:**
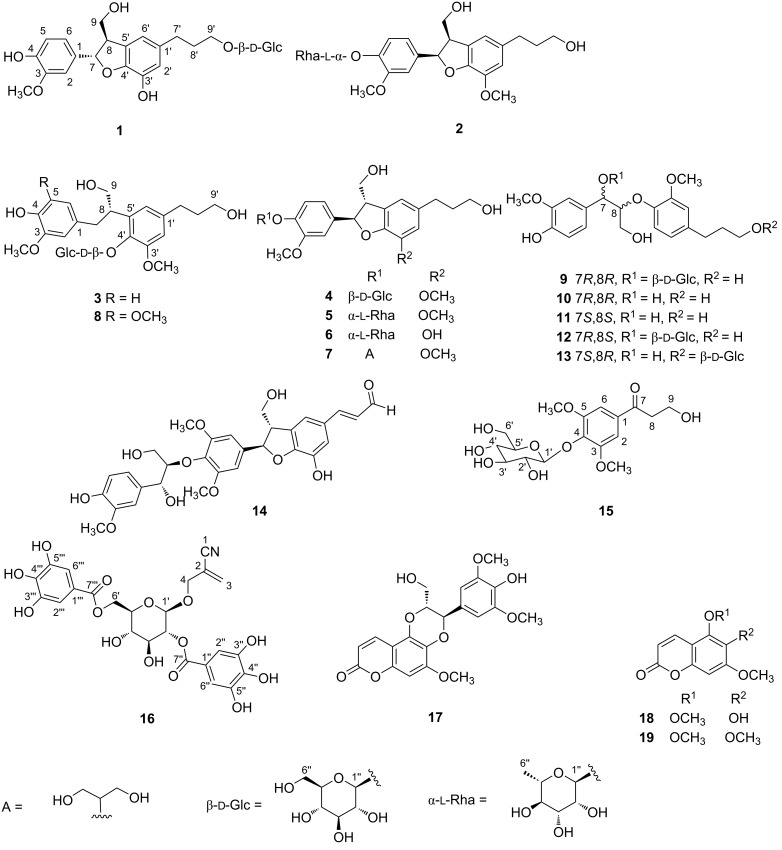
Chemical structures of compounds **1**–**19**.

The MeOH extract of *A*. *fordii* twigs was subjected to liquid–liquid solvent partitioning to yield *n*-hexane, CHCl_3_, EtOAc, and *n*-BuOH-soluble fractions. Repeated column chromatographic purification of the CHCl_3_, EtOAc, and *n*-BuOH-soluble fractions afforded three new neolignan glycosides (**1**–**3**), a new phenolic glycoside (**15**), a new cyanoglucoside (**16**), and 14 known compounds (**4**–**14** and **17**–**19**) (Figure 1).

**Figure 2:**
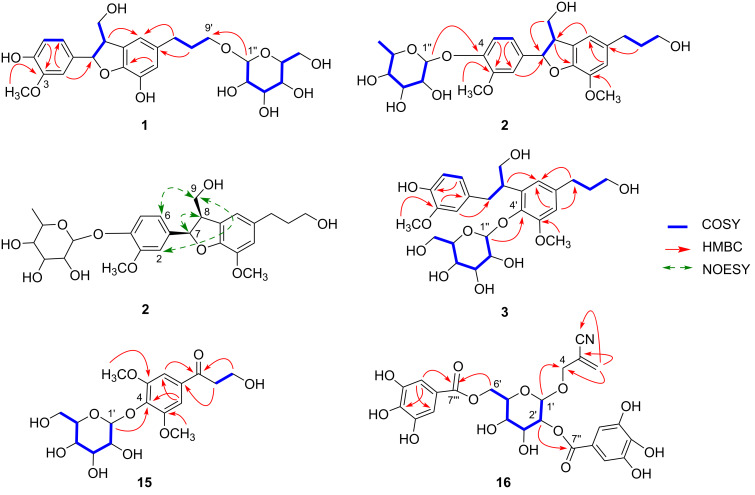
Key COSY and HMBC correlations of compounds **1**–**3**, **15**, and **16** and key NOESY correlation of **2**.

Compound **1** was obtained as a colorless gum. The molecular formula was determined to be C_25_H_32_O_11_ from the [M + Na]^+^ molecular ion peak in the positive mode HRFABMS. The ^1^H NMR data (Table 1) of compound **1** displayed characteristic resonances for a 1,3,4-trisubstituted benzene ring [δ_H_ 7.00 (d, *J* = 1.9 Hz, H-2), 6.87 (dd, *J* = 8.1, 1.9 Hz, H-6), and 6.78 (d, *J* = 8.1 Hz, H-5)], a 1,3,4,5-tetrasubsituted benzene ring [δ_H_ 6.62 (brs, H-2′) and 6.65 (brs, H-6′)], an oxygenated methine [δ_H_ 5.51 (d, *J* = 6.1 Hz, H-7)], an anomeric proton of a sugar [δ_H_ 4.27 (d, *J* = 7.8 Hz, H-1′′)], and a methoxy group [δ_H_ 3.84 (s, 3-OCH_3_)]. The ^13^C NMR data (Table 1) showed 25 peaks including 12 aromatic carbons [δ_C_ 147.6 (C-3), 145.9 (C-4), 145.1 (C-4′), 140.4 (C-3′), 135.2 (C-1′), 133.7 (C-1), 128.3 (C-5′), 118.2 (C-6), 115.8 (C-2′), 115.3 (C-6′), 114.6 (C-5), and 109.1 (C-2)] and six glucose carbons [δ_C_ 103.0 (C-1′′), 76.7 (C-3′′), 76.5 (C-5′′), 73.7 (C-2′′), 70.2 (C-4′′), and 61.3 (C-6′′)]. The spectroscopic data of compound **1** suggested that it is a typical dihydrobenzofuran neolignan glycoside [9–11]. The data for compound **1** were similar to those of glochidioboside isolated from *Glochidion obovatum* [12], except for the presence of a hydroxy group instead of the methoxy group at C-3′ in **1**. The two-dimensional structure of **1** was elucidated via analysis of COSY, HSQC, and HMBC spectroscopic data (Figure 2). The locations of the glucose unit and the methoxy group were confirmed from the observed HMBC correlations of H-1′′/C-9′ and 3-OCH_3_/C-3, respectively (Figure 2). Acid hydrolysis of **1** was conducted to analyze the aglycone and sugar moiety. The structure of the aglycone (**1a**) was confirmed as demethyldihydrodehydrodiconiferyl alcohol based on the comparison of ^1^H NMR and MS data [13]. The relatively large coupling constant of the anomeric proton (7.8 Hz) confirmed that the glucose is combined as β-form [14]. ᴅ-Glucose was identified by co-TLC with a standard sample [CHCl_3_/MeOH/H_2_O 2:1:0.1, *R*_f_ = 0.3)] and GC–MS analysis [15]. The relative configuration at C-7/C-8 of **1** was established as *trans* through the relatively small coupling constant (6.1 Hz) [10,16]. The analysis of the ECD spectrum of **1** determined the absolute configuration of **1** to be 7*S* and 8*R* (positive Cotton effects (CEs) at 292 and 248 nm, and a negative CE at 221 nm; see Supporting Information File 1, Figure S7) [10]. Thus, the structure of compound **1** was elucidated as (7*S*,8*R*)-3′-demethyldihydrodehydrodiconiferyl alcohol 9′-*O*-β-ᴅ-glucopyranoside, and was named as aleuritiside A.

**Table 1:** ^1^H and ^13^C NMR spectroscopic data of compounds **1**–**3** in CD_3_OD.

Pos.	**1** ^a^		**2** ^b^		**3** ^a^	

	δ_C_	δ_H_ (*J* in Hz)	δ_C_	δ_H_ (*J* in Hz)	δ_C_	δ_H_ (*J* in Hz)

1	133.7		134.3		131.9	
2	109.1	7.00, d (1.9)	112.3	7.06, d (2.0)	112.2	6.58, d (2.3)
3	147.6		152		146.9	
4	145.9		146.6		143.9	
5	114.6	6.78, d (8.1)	119.4	7.12, d (8.0)	114.2	6.57 d (8.0)
6	118.2	6.87, dd (8.1, 1.9)	120.1	6.92, dd (8.0, 2.0)	121.2	6.49 dd (8.0, 2.3)
7	87.3	5.51, d (6.1)	88.3	5.85, d (8.8)	37.8	2.99 dd (13.8, 5.5)
						2.72 dd (13.8, 9.6)
8	54.3	3.47, dd (12.6, 6.1)	50.2	3.69, m	41.3	3.98, m
9	63.7	3.85, overlap	63.6	3.31, m	65.6	3.77 dd (10.7, 6.2)
		3.77, dd (11.0, 7.5)				3.68 dd (10.7, 7.3)
1′	135.2		137.1		138.9	
2′	115.8	6.62, br s	114.3	6.76, br s	110.3	6.73, s
3′	140.4		145.3		151.6	
4′	145.1		147.5		142.2	
5′	128.3		131.9		137.1	
6′	115.3	6.65, br s	119.2	6.85, br s	118.9	6.73, s
7’	31.2	2.63, m	33.1	2.65, t (7.5)	31.7	2.66, m
8′	31.5	1.90, dt (13.5, 6.6)	35.9	1.85, m	34.1	1.84, tt (13.0, 6.5)
9′	68.5	3.93, m	62.4	3.59, t (6.5)	60.8	3.59, td (6.5, 1.9)
		3.56, m				
1′′	103.0	4.27 d (7.8)	101.6	5.37, d (1.8)	104.2	4.63 d (7.6)
2′′	73.7	3.22 dd (9.1, 7.8)	72.2	4.08, m	74.5	3.47, m
3′′	76.7	3.37 dd (10.8, 9.1)	72.4	3.89, m	76.4	3.42, m
4′′	70.2	3.30, m	74	3.47, m	69.8	3.39, m
5′′	76.5	3.27 ddd (9.6, 5.6, 2.2)	71	3.83, m	76.6	3.14 ddd (9.2, 5.2, 2.3)
6′′	61.3	3.88 dd (12.0, 2.2)	18.1	1.24, d (6.2)	61.0	3.80 dd (11.0, 8.7)
		3.69 dd (12.0, 5.6)				3.70, overlap
3-OCH_3_	54.9	3.84, s	56.9	3.89, s	54.8	3.71, s
3′-OCH_3_			56.6	3.83, s	54.9	3.82, s

^a^Measured at 700 (δ_H_) and 175 (δ_C_) MHz. ^b^Measured at 500 (δ_H_) and 125 (δ_C_) MHz.

The molecular formula of compound **2**, isolated as a colorless gum, was confirmed to be C_26_H_34_O_10_ from the positive ion mode HRESIMS data. The ^1^H and ^13^C NMR spectra of **2** were very close to that of icariside E_4_ (**5**) [17] with significant differences in the chemical shifts of C-1, C-8, C-9, C-5′, and C-6′ [**2**: δ_C_ 134.3, 50.2, 63.6, 131.9, and 119.2; **5**: δ_C_ 138.9, 55.7, 65.1, 129.7, and 118.0, respectively], indicating that compound **2** could be a stereoisomer of **5** at C-7 and C-8. The inspection of the COSY, HSQC, and HMBC spectra confirmed the planar structure of **2**. The HMBC correlation of H-1′′ to C-4 indicated that the rhamnose unit was linked to the oxygen at C-4 and the characteristic *J* value of the anomeric proton (1.5 Hz) confirmed the rhamnose as α-form (Figure 2) [10]. Acid hydrolysis of compound **2** afforded the aglycone, dihydrodehydrodiconiferyl alcohol (**2a**) [18], and ʟ-rhamnose ([α]_D_^25^ +9.0), which was identified in an identical manner to that of compound **1**. The relatively large coupling constant (8.8 Hz) between H-7 and H-8 in **2**, as opposed to the relatively small coupling constant (6.1 Hz) between H-7 and H-8 in **1**, verified that H-7 and H-8 are *cis*-oriented [10,16], which was supported by the NOESY correlations of H-7/H-8, H-2/H-9, and H-6/H-9 (Figure 2). The ECD spectrum of **2** showed negative CEs at 276 nm and 229 nm and a positive CE at 248 nm, indicating the absolute configuration of C-7 and C-8 as *R* (Figure S15 in Supporting Information File 1) [19]. Therefore, the structure of compound **2** was determined to be (7*R*,8*R*)-dihydrodehydrodiconiferyl alcohol 4-*O*-α*-*ʟ-rhamnopyranoside and was named as aleuritiside B.

Compound **3** was obtained as a colorless gum after purification with a molecular formula of C_26_H_36_O_11_ as deduced from the positive molecular ion peak [M + Na]^+^ at *m/z* 547.2155 (calcd for C_26_H_36_O_11_Na, 547.2155) in the HRESIMS. Analysis of the ^1^H and ^13^C NMR data (Table 1) showed a 1,3,4-trisubstituted benzene ring [δ_H_ 6.58 (d, *J* = 2.3 Hz, H-2), 6.57 (d, *J* = 8.0 Hz, H-5), and 6.49 (dd, *J* = 8.0, 2.3 Hz, H-6); δ_C_ 146.9 (C-3), 143.9 (C-4), 131.9 (C-1), 121.2 (C-6), 114.2 (C-5), and 112.2 (C-2)], a 1,3,4,5-tetrasubstituted benzene ring [δ_H_ 6.73 (s, H-2′, 6′); *δ*_C_ 151.6 (C-3′), 142.2 (C-4′), 138.9 (C-1′), 137.1 (C-5′), 118.9 (C-6′), and 110.3 (C-2′)], a glucopyranose unit [δ_H_ 4.63 (d, *J* = 7.6 Hz, H-1′′); δ_C_ 104.2 (C-1′′), 76.6 (C-5′′), 76.4 (C-3′′), 74.5 (C-2′′), 69.8 (C-4′′), and 61.0 (C-6′′)], and two methoxy groups [δ_H_ 3.82 (s, 3′-OCH_3_) and 3.71 (s, 3-OCH_3_); δ_C_ 54.9 (s, 3′-OCH_3_) and 54.8 (s, 3-OCH_3_)]. The spectroscopic data resembled closely to those of icariside E_3_, isolated from *Epimedium grandiflorum* var. *thunbergianum* [20], indicating that compound **3** may have the identical planar structure to icariside E_3_, which was reported without assignment of the absolute configuration. The planar structure of **3** was further confirmed by analysis of 2D NMR data, including COSY, HSQC, and HMBC (Figure 2). The determination of the stereochemistry for the sugar unit of **3** was conducted following the same method as for compound **2**. The structure of the aglycone **3a** obtained by acid hydrolysis of **3** was confirmed based on ^1^H NMR and MS data [20]. The absolute configuration of **3a** was established as 8*S* (a negative CE at 273 nm) based on the comparison of its ECD spectrum with the reported data [21]. Thus, the structure of compound **3** was determined as 8*S*-tetrahydrodehydrodiconiferyl alcohol 4-*O*-α*-*ʟ-rhamnopyranoside and was named aleuritiside C.

Compound **15** was obtained as a yellow gum. The [M + Na]^+^ ion peak at *m*/*z* 411.1260 (calcd for 411.1267) in the HRESIMS corresponded to the molecular formula C_17_H_24_O_10_. The IR spectrum exhibited signals at 3321 cm^−1^ and 1675 cm^−1^ suggesting the presence of hydroxy and carbonyl groups, respectively. The ^1^H NMR spectrum of compound **15** (Table 2) exhibited signals for a 1,3,4,5-tetrasubstituted aromatic ring [δ_H_ 7.23 (s, 2H, H-2 and H-6)], two methoxy groups [δ_H_ 3.81 (s, 6H, 3,5-OCH_3_)], an anomeric proton [δ_H_ 5.00 (d, *J* = 7.7 Hz, 1H, H-1′)], and two methylenes [δ_H_ 3.23 (t, *J* = 6.0 Hz, 2H, H-8) and 3.96 (t, *J* = 6.0 Hz, 2H, H-9)]. The ^13^C NMR spectrum of compound **15** (Table 2) revealed 14 peaks for 17 carbons including a ketone carbon (δ_C_ 200.0), a 1,3,4,5-tetrasubstituted aromatic ring [δ_C_ 154.4 (×2), 140.7, 134.6, and 107.5 (×2)], two methoxy groups [57.3 (×2)], and a glucose unit (δ_C_ 104.6, 78.6, 78.1, 75.8, 71.5, and 62.7). The location of the glucose unit was determined to be at C-4 by analysis of the HMBC data showing a correlation from H-1′ to C-4. The coupling constant (7.7 Hz) of the anomeric proton of glucose suggested that it was the β-form. Acid hydrolysis of **15** yielded 3-hydroxy-1-(4-hydroxy-3,5-dimethoxyphenyl)-1-propanone (**15a**), whose ^1^H NMR spectral data were in good agreement with the values reported previously [22], and ᴅ-glucopyranose was identified through co-TLC and the specific rotation value {[α]_D_^25^ +86.0 (*c* 0.03, MeOH)}. Accordingly, the structure of compound **15** was identified as 2,6-dimethoxy-4-(1-oxo-3-hydroxypropyl)phenyl β-ᴅ-glucopyranoside and named aleuriteoside A.

**Table 2:** ^1^H and ^13^C NMR spectral data of **15** and **16** in CD_3_OD.

pos.	**15** ^a^		**16** ^b^	

	δ_C_	δ_H_ (*J* in Hz)	δ_C_	δ_H_ (*J* in Hz)

1	134.6		121.3	
2	107.5	7.23, s	118.2	
3	154.4		133.2	5.97, br s
				5.95, br s
4	140.7		69.3	4.34, dd (13.7, 1.5)
				4.22, dd (13.7, 1.5)
5	154.4			
6	107.5	7.23, s		
7	200.0			
8	42.1	3.23, t (6.0)		
9	58.8	3.96, t (6.0)		
1′	104.6	5.00, d (7.7)	102.1	4.70, d (8.0)
2′	75.8	3.51, m	75.2	4.99, dd (9.5, 8.0)
3′	78.6	3.23, m	76.0	3.70, m
4′	71.5	3.42, m	71.8	3.57, m
5′	78.1	3.44, m	76.2	3.67, m
6′	62.7	3.76, dd (12.0, 2.0)	64.5	4.57, dd (12.0, 2.0)
		3.66, dd (12.0, 5.0)		4.46, dd (12.0, 5.0)
1′′			121.4	
2′′,6′′			110.6	7.10 s
3′′,5′′			146.6	
4′′			140.1	
7′′			167.7	
1′′′			121.4	
2′′′,6′′′			110.3	7.11s
3′′′,5′′′			146.7	
4′′′			140.1	
7′′′			168.4	
3,5-OCH_3_	57.3	3.81, s		

^a^Measured at 700 (δ_H_) and 175 (δ_C_) MHz. ^b^Measured at 500 (δ_H_) and 125 (δ_C_) MHz.

Compound **16** was isolated as a colorless gum and the molecular formula was determined to be C_24_H_24_NO_14_ from the [M + Na]^+^ ion in the positive ion HRESIMS. The ^1^H NMR spectrum of **16** (Table 2) showed two 1,3,4,5-tetrasubstituted aromatic rings [δ_H_ 7.10 (s, 2H, H-2′′ and H-6′′), and 7.11 (s, 2H, H-2′′′ and H-6′′′)], a terminal olefinic methylene [δ_H_ 5.97 (br s, 1H, H-3a), and 5.95 (br s, 1H, H-3b)], two oxygenated methylenes [δ_H_ 4.34 (dt, *J* = 13.7, 1.5 Hz,1H, H-4a) and 4.22 (dt, *J* = 13.7, 1.5 Hz, 1H, H-4b); 4.57 (dd, *J* = 12.0, 2.0 Hz, 1H, H-6′a) and 4.46 (dd, *J* = 12.0, 5.0 Hz, 1H, H-6′b)], and an anomeric proton [δ_H_ 4.70 (d, *J* = 8.0 Hz, 1H, H-1′)]. The ^13^C NMR spectrum of **16** (Table 2) revealed 24 carbons, including two galloyl moieties [δ_C_ 168.4, 167.7, 146.7 (×2), 146.6 (×2), 140.1 (×2), 121.4 (×2), 110.6 (×2), and 110.3 (×2)], a glucose moiety (δ_C_ 102.1, 76.2, 76.0, 75.2, 71.8, and 64.5), and 2-(hydroxymethyl)acrylonitrile signals (δ_C_ 133.2, 121.3, 118.2, and 69.3). 1D NMR spectra of **16** were similar to those of taxilluside C [23], except for the presence of a 2-(hydroxymethyl)acrylonitrile moiety at C-1′ instead of 1,1-dimethylallyl alcohol. Furthermore, the NMR signals of cyanoglucoside moiety of **16** were in good agreement with literature data reported from *Codiaeum variegatum* belonging to the same family [24]. The location of the glucose unit was determined to be at C-4 based on the analysis of the HMBC data showing a correlation from H-1′ to C-4 (Figure 2). The HMBC cross-peaks of H-2′/C-7′′ and H-6′/C-7′′′ also indicated the presence of two galloyl groups at C-2′ and C-6′ of the glucose unit, respectively (Figure 2). Alkaline hydrolysis of **16** yielded codiacyanoglucoside (**16a**) and gallic acid (**16b**). The identification of **16a** and **16b** was conducted by comparison of their ^1^H NMR and MS data [24–25]. Consequently, the structure of **16** was determined to be codiacyano glucosyl-2′,6′-O-digallate, named aleucyanoglucoside.

The other known compounds were identified as 7*R*,8*S*-dihydrodehydrodiconiferyl alcohol 4-*O*-β-ᴅ-glucopyranoside (**4**) [26], icariside E_4_ (**5**) [17], isomassonianoside B (**6**) [27], sakuraresinol (**7**) [28], selaginellol 4′-*O*-β-ᴅ-glucopyranoside (**8**) [21], 7*R*,8*R*-4,7,9,9′-tetrahydroxy-3,3′-dimethoxy-8-*O*-4′-neolignan-7-*O*-β-ᴅ-glucopyranoside (**9**) [29], 7*R*,8*R*-4,9,9′-trihydroxy-3,3′-dimethoxy-8-*O*-4′-neolignan (**10**) [30], 7*S*,8*S*-4,9,9′-trihydroxy-3,3′-dimethoxy-8-*O*-4′-neolignan (**11**) [30], 7*R*,8*S*-4,7,9,9′-tetrahydroxy-3,3′-dimethoxy-8-*O*-4′-neolignan-7-*O*-β-ᴅ-glucopyranoside (**12**) [29], 7*S*,8*R*-4,7,9-trihydroxy-3,3′-dimethoxy-8-*O*-4′-neolignan-9′-*O*-β-ᴅ-glucopyranoside (**13**) [31], buddlenol A (**14**) [32], aleuritin (**17**) [33], fraxinol (**18**) [34], and 5,6,7-trimethoxycoumarin (**19**) [34] based on the comparison of their spectroscopic data and specific rotation with the reported data.

Compounds **1**–**19** were tested for their effects on nitric oxide (NO) production levels in lipopolysaccharide (LPS)-stimulated murine microglial BV-2 cells to evaluate for antineuroinflammatory activities (Table 3). Compound **14** showed relative inhibitory effects on NO production with an IC_50_ value of 20.9 μM which was stronger than the positive control (L-NMMA, IC_50_ 28.8 μM). Compounds **11**, **17**, and **19** also displayed moderate activity (IC_50_ 35.5–37.1 μM), whereas compounds **2**, **4**, **10**, **12**, and **13** exhibited only weak effects (IC_50_ 42.1–55.0 μM). Interestingly, compounds **2** and **5** have the same planar structures with only differing in the C-7 stereochemistry, but they showed quite different inhibition effects on NO production (IC_50_ 55.0 μM, **2**; IC_50_ > 500 μM, **5**). The MTT cell viability test suggested that all the compounds had no cytotoxic effect on BV-2 cell survival at a concentration of 20 μM.

**Table 3:** Effects of isolated compounds on NO production in LPS-activated BV-2 cells.

comp.	IC_50_ (µM)^a^	cell viability%^b^	comp.	IC_50_ (µM)^a^	cell viability%^b^

**1**	>500	87.5 ± 5.1	**11**	37.1	89.97 ± 3.2
**2**	55.0	90.3 ± 3.1	**12**	47.6	90.1 ± 3.7
**3**	109.8	93.1 ± 3.5	**13**	42.1	87.9 ± 4.2
**4**	48.6	86.9 ± 6.5	**14**	20.9	105.2 ± 1.5
**5**	>500	111.9 ± 4.3	**15**	93.7	93.3 ± 7.4
**6**	74.6	86.2 ± 6.2	**16**	>500	95.8 ± 4.2
**7**	278.8	90.2 ± 4.1	**17**	35.5	101.8 ± 4.2
**8**	126.6	87.9 ± 5.1	**18**	117.0	96.6 ± 4.4
**9**	321.7	87.9 ± 7.6	**19**	36.7	99.7 ± 2.9
**10**	42.9	86.3 ± 4.4	L-NMMA^c^	28.8	99.9 ± 3.6

^a^The IC_50_ value of each compound was defined as the concentration (μM) that caused 50% inhibition of NO production in LPS-activated BV-2 cells. ^b^The cell viability after treatment with 20 μM of each compound was measured using the MTT assay and is expressed as a percentage (%). Results are the means of three independent experiments, and data are expressed as the means ± SD. ^c^Positive control substance.

Compounds **1**–**19** were also tested for their neuroprotection activity by measuring the secretion of NGF from C6 cells into the medium (Table 4). Compounds **8** and **16** stimulated NGF release, exhibiting stimulation levels of 134.2 ± 8.1% and 134.6 ± 5.9%, respectively. Although compounds **3** and **8** have similar structures without or with a methoxy group at C-5, respectively, only compound **8** showed a significant activity (96.2 ± 1.1% for **3**). The other compounds exhibited moderate or no NGF secretion effect.

**Table 4:** Effects of isolated compounds on NGF secretion in C6 cells.

comp.	NGF secretion^a^ (%)	cell viability^b^ (%)	comp.	NGF secretion^a^ (%)	cell viability^b^ (%)

**1**	117.4 ± 2.8	92.7 ± 1.3	**11**	102.1 ± 5.4	92.3 ± 0.3
**2**	101.3 ± 7.6	90.5 ± 0.1	**12**	110.3 ± 0.8	94.0 ± 1.9
**3**	96.2 ± 1.1	91.0 ± 0.8	**13**	104.5 ± 3.2	92.9 ± 0.2
**4**	99.1 ± 1.0	93.9 ± 1.1	**14**	12.0 ± 0.4	99.6 ± 3.1
**5**	102.5 ± 8.0	91.1 ± 2.0	**15**	111.3 ± 7.6	98.0 ± 4.1
**6**	113.9 ± 0.9	93.0 ± 0.5	**16**	134.6 ± 5.9	88.8 ± 3.9
**7**	101.2 ± 5.8	97.9 ± 0.3	**17**	102.1 ± 2.5	98.7 ± 2.7
**8**	134.2 ± 8.1	94.5 ± 3.9	**18**	99.2 ± 2.7	97.9 ± 4.3
**9**	121.0 ± 0.6	94.4 ± 1.8	**19**	104.1 ± 4.6	98.7 ± 3.7
**10**	109.0 ± 5.3	92.9 ±4.1	6-shogaol^c^	143.9 ± 12.5	95.6 ± 1.8

^a^C6 cells were treated with 20 μM of each test compound. After 24 h, the content of NGF secreted in the C6-conditioned medium was measured by ELISA. The level of secreted NGF is expressed as the percentage of the untreated control (set as 100%). ^b^Cell viability after treatment with 20 μM of each compound was determined by an MTT assay and is expressed as a percentage (%). Results are the means of three independent experiments, and the data are expressed as means ± SD. ^c^Positive control substance.

The cytotoxicity of compounds **1**–**19** was also evaluated against four human cancer cell lines (A549, SK-OV-3, SK-MEL-2, and HCT-15) through an SRB assay. All the tested compounds showed no activity for the cell lines (IC_50_ > 10 μM).

## Conclusion

Isolation of phytochemical constituents from the twigs of *A. fordii* led to the discovery of three new neolignan glycosides **1**–**3**, a new phenolic glycoside **15**, and a new cyanoglycoside **16** along with 14 known compounds **4**–**14** and **17**–**19**. The structural characterization of the new compounds was conducted based on the analysis of their spectroscopic and spectrometric data, and chemical methods. All isolated compounds were tested for their antineuroinflammatory and neuroprotective activities. Compound **14** showed inhibition effects on NO production and the stereoisomers **2** and **5** demonstrated the difference in activity according to the configuration. Compounds **8** and **16** exhibited neuroprotection effects. Thus, this study indicates that the active phenolic compounds from *A. fordii* would be potential candidates for drug discovery associated with antineurodegenerative diseases.

## Experimental

**General experimental procedures**. Optical rotations were measured on a JASCO P-2000 polarimeter. IR spectra were acquired with a JASCO FT/IR-4600 spectrometer. UV spectra were obtained on a Shimadzu UV-1601 UV–visible spectrophotometer. NMR spectra were recorded on a Bruker AVANCE III 700 NMR spectrometer operating at 700 MHz (^1^H) and 175 MHz (^13^C) with chemical shifts given in ppm (δ) and a Varian UNITY INOVA 500 NMR spectrometer (Varian Palo Alto, CA, USA) operating at 500 MHz (^1^H) and 125 MHz (^13^C). HRESIMS spectra were obtained on a Waters SYNAPT G2 mass spectrometer and semipreparative HPLC was conducted using a Gilson 306 pump with a Shodex refractive index detector and a Phenomenex Luna 10 μm column (250 × 10 mm). Silica gel 60 (Merck, Darmstadt, 70–230 mesh, and 230–400 mesh) and RP-C_18_ silica gel (Merck, 230–400 mesh) were used for column chromatography. Low-pressure liquid chromatography was performed over Merck LiChroprep Lobar-A Si gel 60 (240 × 10 mm) with an FMI QSY-0 pump (ISCO). Merck precoated silica gel F_254_ plates and RP-18 F_254s_ plates were used for TLC. Spots were detected on TLC under UV light or by heating after spraying the samples with anisaldehyde-sulfuric acid.

**Plant material.** Twigs of *A. fordii* were collected in Chungbuk Goesan, Korea in August 2012 and the plant was identified by Dr. Kang Ro Lee, Professor at Sungkyunkwan University. A voucher specimen (SKKU-NPL 1212) has been deposited in the herbarium of the School of Pharmacy, Sungkyunkwan University, Suwon, Republic of Korea.

**Extraction and isolation.** Twigs of *A. fordii* (7.0 kg) were extracted three times with 80% aqueous MeOH (each 10 L × 1 day) under reflux and filtered. The filtrate was evaporated under vacuum to obtain a crude MeOH extract (325 g), which was suspended in distilled water and successively partitioned with *n*-hexane, CHCl_3_, EtOAc, and *n*-BuOH, to yield 15 g, 15 g, 9 g, and 23 g of each residue, respectively. The CHCl_3_-soluble layer (15.0 g) was separated by Sephadex LH-20 chromatography (80% aq. MeOH) to yield six fractions (C1–C6). Fraction C1 (2.0 g) was subjected to RP-C_18_ silica gel chromatography, eluting with gradient solvent system (30 → 100% aq. MeOH) to yield four subfractions (C2A–C2D). Fraction C2C (210 mg) was purified by semipreparative HPLC (2 mL/min, 50% aq. MeOH) to yield compounds **17** (5 mg) and **19** (6 mg). Fraction C3 (5.2 g) was subjected to RP-C_18_ silica gel chromatography, eluting with gradient solvent system (30 → 100% aq. MeOH) to yield five subfractions (C3A–C3E). Fraction C3B (554 mg) was subjected to repeated RP-C_18_ silica gel chromatography and further purified by semipreparative HPLC (50% aq. MeOH) to yield compound **18** (8 mg). Fraction C3D (1.0 g) was subjected to repeated RP-C_18_ silica gel chromatography and further purified by semipreparative HPLC (23% aq. CH_3_CN) to yield compound **14** (5 mg). Fraction C4 (0.5 g) was subjected to RP-C_18_ silica gel chromatography, eluting with gradient solvent system (30 → 100% aq. MeOH) to yield six subfractions (C4A–C4F). Compounds **7** (4 mg), **10** (4 mg), and **11** (4 mg) were obtained by purification of fraction C4D (43 mg) and C4E (57 mg) using semipreparative HPLC (15% aq. CH_3_CN).

The EtOAc-soluble layer (9.0 g) was separated on a silica gel column (CHCl_3_/MeOH 15:1 → 1:1) to yield eight fractions (E1–E8). Fraction E7 (0.4 g) was subjected to RP-C_18_ silica gel chromatography, eluting with gradient solvent system (30 → 100% aq. MeOH) to yield nine subfractions (E7A–E7I). Fractions E7A (31 mg), E7B (98 mg), E7C (30 mg), and E7D (37 mg) were purified by semipreparative HPLC (15% aq. MeOH and 25–30 % aq. CH_3_CN) to yield compounds **2** (3 mg), **5** (6 mg), and **15** (3 mg). Fraction E8 (0.9 g) was subjected to RP-C_18_ silica gel chromatography, eluting with gradient solvent system (30 → 100% aq. MeOH) to yield six subfractions (E8A–E8F). Compounds **4** (4 mg), **6** (3 mg), and **16** (4 mg) were obtained by purification of fractions E8B (54 mg) and E8C (120 mg) using semipreparative HPLC (40% aq. MeOH).

The *n*-BuOH-soluble layer (23.0 g) was chromatographed on a Diaion HP-20 column, eluting with an isocratic solvent system of 100% H_2_O and 100% MeOH, yielding H_2_O and MeOH-soluble fractions. The MeOH fraction was subjected to separation on a silica gel column (CHCl_3_/MeOH/H_2_O 6:1:0.1 → 1:1:0.1) to afford five fractions (BM1–BM5). Fraction BM3 (2.5 g) was fractionated over an RP-C_18_ silica gel column, eluting with gradient solvent system (25 → 100% aq. MeOH) to give nine subfractions (BM3A–BM3I). Subfraction BM3E (61 mg) was purified by semipreparative HPLC (23% aq. CH_3_CN) to acquire compound **13** (3 mg). Compounds **3** (8 mg) and **8** (8 mg) were isolated upon purification of subfraction BM3G (52 mg) by semipreparative HPLC (17% aq. CH_3_CN). Fraction BM4 (1.0 g) was subjected to passage over an RP-C_18_ silica gel column, eluting with gradient solvent system (15 → 100% aq. MeOH) to acquire 17 subfractions (BM4A–BM4Q). Compounds **1** (5 mg) and **12** (9 mg) were obtained by purification of fraction BM4M (65 mg) using semipreparative HPLC (15% aq. CH_3_CN). Fraction BM4N (58 mg) was purified by semipreparative HPLC (30% aq. CH_3_CN) to yield compound **9** (10 mg).

**Aleuritiside A (1).** Colorless gum; [α]_D_^25^ −12.1 (*c* 0.05, MeOH); IR (KBr) *ν*_max_: 3360, 2943, 2830, 1448, 1033 cm^−1^; UV (MeOH) λ_max_, nm (log ε): 282 (1.40), 228 (3.61); ECD (MeOH) λ_max_, nm (Δε): 292 (5.3), 248 (3.3), 221 (−2.1); ^1^H and ^13^C NMR data, see Table 1; positive HRMS–FAB (*m/z*): [M + Na]^+^ calcd for C_25_H_32_O_11_Na, 531.1837; found, 531.1844.

**Aleuritiside B (2).** Colorless gum; [α]_D_^25^ −15.4 (*c* 0.05, MeOH); IR (KBr) *ν*_max_: 3355, 2945, 2832, 1453, 1033 cm^−1^; UV (MeOH) λ_max_, nm (log ε): 283 (1.31), 230 (3.53); ECD (MeOH) λ_max_, nm (Δε): 276 (−3.3), 248 (5.1), 229 (−8.5) nm; ^1^H and ^13^C NMR data, see Table 1; positive HRMS–ESI (*m/z*): [M + Na]^+^ calcd for C_26_H_34_O_10_Na, 529.2050; found, 529.2050.

**Aleuritiside C (3).** Colorless gum; [α]_D_^25^ −23.4 (*c* 0.05, MeOH); IR (KBr) *ν*_max_: 3361, 2946, 2830, 1462, 1029 cm^−1^; UV (MeOH) λ_max_, nm (log ε): 275 (2.53); ECD (MeOH) λ_max_, nm (Δε): 273 (−8.1), 236 (−8.3); ^1^H and ^13^C NMR data, see Table 1; positive HRMS–ESI (*m/z*): [M + Na]^+^ calcd for C_26_H_36_O_11_Na, 547.2155; found, 547.2155.

**Aleuriteoside A (15).** Colorless gum; [α]_D_^25^ −13.7 (*c* 0.08, MeOH); IR (KBr) *ν*_max_: 3321, 2975, 1675, 1601, 1453, 1065 cm^−1^; UV (MeOH) λ_max_, nm (log ε): 280 (2.31); ^1^H and ^13^C NMR data, see Table 2; positive HRMS–ESI (*m/z*): [M + Na]^+^ calcd for C_17_H_24_O_10_Na, 411.1267; found, 411.1260.

**Aleucyanoglucoside (16).** Colorless gum; [α]_D_^25^ −33.5 (*c* 0.30, MeOH); IR (KBr) *ν*_max_: 3535, 3330, 2832, 2218, 1453, 1033 cm^−1^; UV (MeOH) λ_max_, nm (log ε): 283 (1.31), 230 (3.53); ^1^H and ^13^C NMR data, see Table 2; positive HRMS–ESI (*m/z*): [M + Na]^+^ calcd for C_24_H_24_NO_14_Na, 572.1016; found, 572.1014.

**Acid hydrolysis and sugar analysis.** In a manner similar as described in [35], compounds **1**–**3** and **15** (each 1.0**–**2.0 mg) were refluxed with 1 mL of 1 N HCl for 1 h at 90 °C. The hydrolysate was extracted with EtOAc and the aqueous layer was neutralized by passing it through an Amberlite IRA-67 column to give the sugar. The sugar obtained from the hydrolysis was dissolved in anhydrous pyridine (0.5 mL) followed by adding of ʟ-cysteine methyl ester hydrochloride (2.0 mg, Sigma-Aldrich, St. Louis, MO, USA). The mixture was stirred at 60 °C for 1.5 h and trimethylsilylated with 1-trimethylsilylimidazole (0.1 mL, Sigma-Aldrich, St. Louis, MO, USA) for 2 h. The mixture was partitioned between *n*-hexane and H_2_O (1.0 mL each), and the organic layer (1.0 μL) was analyzed by GC–MS. The identification of ᴅ-glucose and ʟ-rhamnose was carried out by coinjection of the hydrolysate with standard silylated samples, giving a single peak at 9.730 and 9.712 min, respectively. Authentic samples (Sigma-Aldrich, St. Louis, MO, USA) that were treated in the same way showed a single peak at 9.730 and 9.708 min, respectively.

**Alkaline hydrolysis of 16.** In a manner similar as described in [36], compound **16** (1.0 mg) was hydrolyzed with 0.1 N KOH (1 mL) at room temperature for 4 h. The reaction mixture was subsequently passed through an ion exchange column (Dowex^®^ 50WX8 hydrogen form, Sigma-Aldrich, St. Louis, MO, USA) using deionized water to remove KOH. A portion of the reaction product was partitioned between EtOAc/H_2_O (each 1.0 mL) and the aglycone **16a** was acquired from the EtOAc-soluble phase.

**Measurement of nitric oxide production and cell viability.** In a manner similar as described in [37], BV2 cells were used to test the inhibitory effect of the isolated compounds on LPS-stimulated NO production [38–39]. The BV2 cells seeded on a 96-well plate (4 × 10^4^ cells/well) were treated with and without various concentrations of the test compounds. The treated cells were stimulated with LPS (100 ng/mL) and incubated for 24 h. The level of nitrite (NO_2_, a soluble oxidation product of NO) in the culture medium was measured using the Griess reagent (0.1% *N*-1-naphthylethylenediamine dihydrochloride and 1% sulfanilamide in 5% phosphoric acid). The supernatant (50 μL) in each well was harvested and mixed with an equal volume of Griess reagent. After 10 min, the absorbance was measured at 570 nm with a microplate reader (Emax, Molecular Devices, Sunnyvale, CA, USA). Graded sodium nitrite solution was used as a standard to gauge NO_2_ concentration. Cell viability was assessed by the MTT assay.

**Measurement of NGF secretion and cell viability assays.** C6 glioma cells (Korean Cell Line Bank, Seoul, Republic of Korea) were used to measure the release of nerve growth factor (NGF) into the culture medium. The C6 cells were seeded onto 24-well plates at a density of 1 × 10^5^ cells/well. After 24 h, the cells were treated with serum-free DMEM and incubated with different concentrations of the test compounds for an additional 24 h. The NGF levels were evaluated in the medium supernatant using an ELISA development kit. Cell viability was measured using the MTT assay and the results were expressed as a percentage of the control group (untreated cells).

**Cytotoxicity assessment.** The SRB assay was performed to evaluate cytotoxicity of all the isolated compounds against four cultured human cancer cell lines. The cell lines (National Cancer Institute, Bethesda, MD, USA) were used A549 (non-small cell lung adenocarcinoma), SK-OV-3 (ovarian malignant ascites), SK-MEL-2 (skin melanoma), and HCT-15 (colon adenocarcinoma). Each cell line was inoculated over standard 96-well flat-bottom microplates and incubated for 24 h at 37 °C in condition of a humidified atmosphere of 5% CO_2_. The attached cells were incubated with various concentrations of the test compounds and the cells were cultured for 48 h. Then, the culture medium was removed from each well and the cells were fixed with 10% cold trichloroacetic acid at 4 °C for 1 h. After the supernatant was discarded and the plates were washed with tap water, the cells were stained with 0.4% SRB solution and incubated for 30 min at room temperature. These cells were washed to remove the unbound dye and subsequently solubilized with 10 mM unbuffered Tris base solution (pH 10.5). The absorbance was measured spectrophotometrically at 520 nm with a microtiter plate reader. Etoposide (Sigma Chemical Co., ≥98%) was used as a positive control.

## Supporting Information

File 1 Copies of NMR spectra including 1D and 2D NMR and HRMS data of compounds **1**–**3**, **15**, and **16** and ECD spectra of compounds **1**–**3**.
