# Supplementary material for: Phenolic constituents from twigs of Aleurites fordii and their biological activities
Source: Beilstein J Org Chem. 2021 Sep 7;17:2329–39. doi: 10.3762/bjoc.17.151 (PMC8450942; doi:10.3762/bjoc.17.151)
Supplement: File 1 — Copies of NMR spectra including 1D and 2D NMR and HRMS data of compounds 1–3, 15, and 16 and ECD spectra of compounds 1–3. [file Beilstein_J_Org_Chem-17-2329-s001.pdf]

BEILSTEIN JOURNAL OF ORGANIC CHEMISTRY

Supporting Information

for

Phenolic constituents from twigs of *Aleurites fordii* and their biological activities

Kyoung Jin Park, Won Se Suh, Da Hye Yoon, Chung Sub Kim, Sun Yeou Kim and Kang Ro Lee

Beilstein J. Org. Chem. **2021,** *17,* 2329–2339. doi:10.3762/bjoc.17.151

Copies of NMR spectra including 1D and 2D NMR and HRMS data of compounds 1–3, 15, and 16 and ECD spectra of compounds 1–3

License and Terms: This is a supporting information file under the terms of the Creative Commons Attribution License (https://creativecommons.org/licenses/by/4.0). Please note that the reuse, redistribution and reproduction in particular requires that the author(s) and source are credited and that individual graphics may be subject to special legal provisions.
The license is subject to the *Beilstein Journal of Organic Chemistry* terms and conditions: (https://www.beilstein-journals.org/bjoc/terms)

Contents:

Figure S1 HRFABMS data of **1**

Figure S2 ^{1}H NMR data of **1** (CD_3OD, 700 MHz)

Figure S3 ^{13}C NMR data of **1** (CD_3OD, 175 MHz)

Figure S4 ^{1}H-^{1}H COSY data of **1** (CD_3OD)

Figure S5 HSQC data of **1** (CD_3OD)

Figure S6 HMBC data of **1** (CD_3OD)

Figure S7 ECD data of **1**

Figure S8 HRESIMS data of **2**

Figure S9 ^{1}H NMR data of **2** (CD_3OD, 500 MHz)

Figure S10 ^{13}C NMR data of **2** (CD_3OD, 125 MHz)

Figure S11 ^{1}H-^{1}H COSY data of **2** (CD_3OD)

Figure S12 HSQC data of **2** (CD_3OD)

Figure S13 HMBC data of **2** (CD_3OD)

Figure S14 NOESY data of **2** (CD_3OD)

Figure S15 ECD data of **2**

Figure S16 HRESIMS data of **3**

Figure S17 ^{1}H NMR data of **3** (CD_3OD, 700 MHz)

Figure S18 ^{13}C NMR data of **3** (CD_3OD, 175 MHz)

Figure S19 ^{1}H-^{1}H COSY data of **3** (CD_3OD)

Figure S20 HSQC data of **3** (CD_3OD)

Figure S21 HMBC data of **3** (CD_3OD)

Figure S22 ECD data of **3**

Figure S23 HRESIMS data of **15**

Figure S24 ^{1}H NMR data of **15** (CD_3OD, 700 MHz)

Figure S25 ^{13}C NMR data of **15** (CD_3OD, 175 MHz)

Figure S26 ^{1}H-^{1}H COSY data of **15** (CD_3OD)

Figure S27 HSQC data of **15** (CD_3OD)

Figure S28 HMBC data of **15** (CD_3OD)

Figure S29 HRESIMS data of **16**

Figure S30 ^{1}H NMR data of **16** (CD_3OD, 500 MHz)

Figure S31^{13}C NMR data of **16** (CD_3OD, 125 MHz)

Figure S32 ^{1}H-^{1}H COSY data of **16** (CD_3OD)

Figure S33 HSQC data of **16** (CD_3OD)

Figure S34 HMBC data of **16** (CD_3OD)

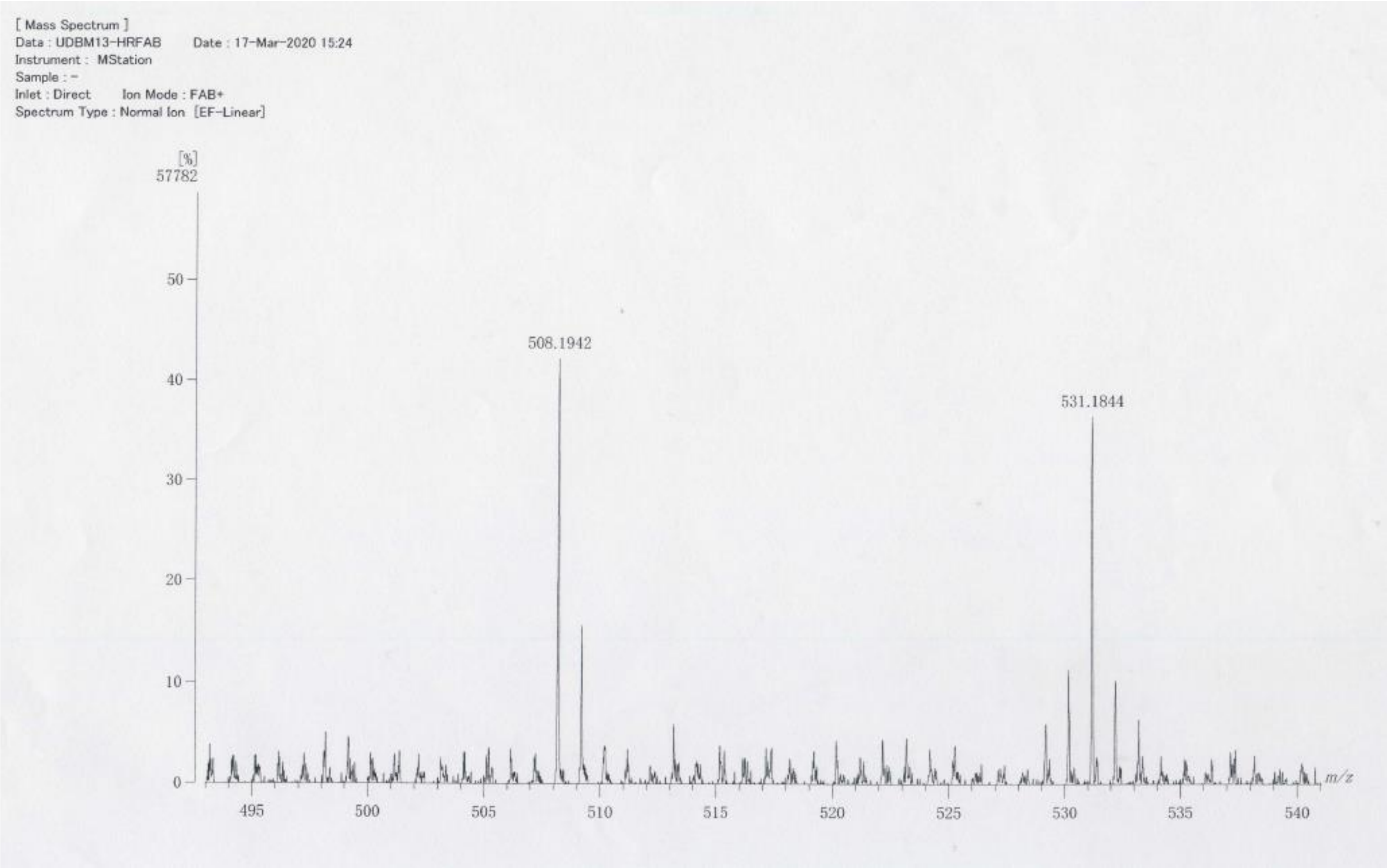

Figure S1 HRFABMS data of **1**

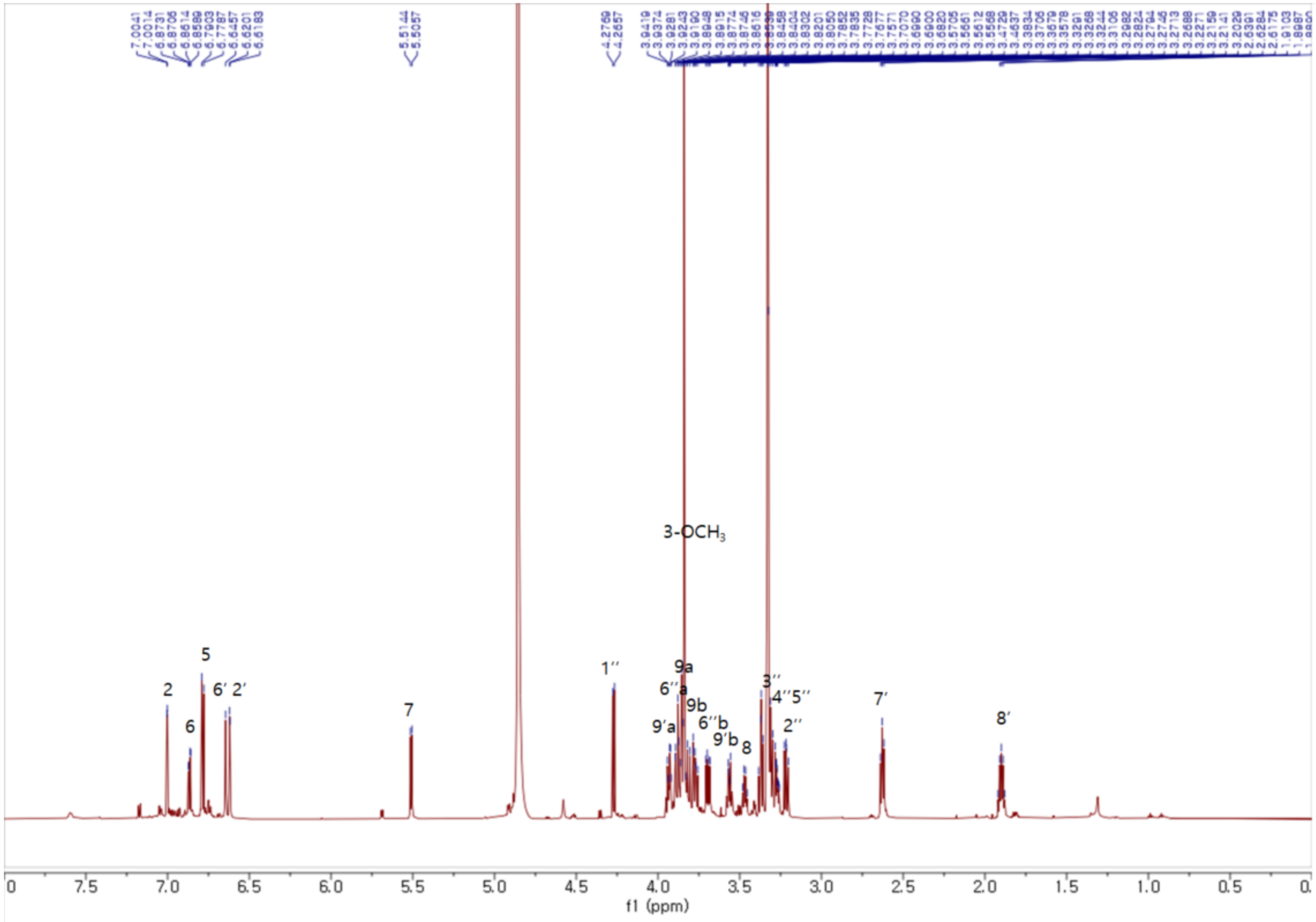

Figure S2 ^{1}H NMR data of **1** (CD_3OD, 700 MHz)

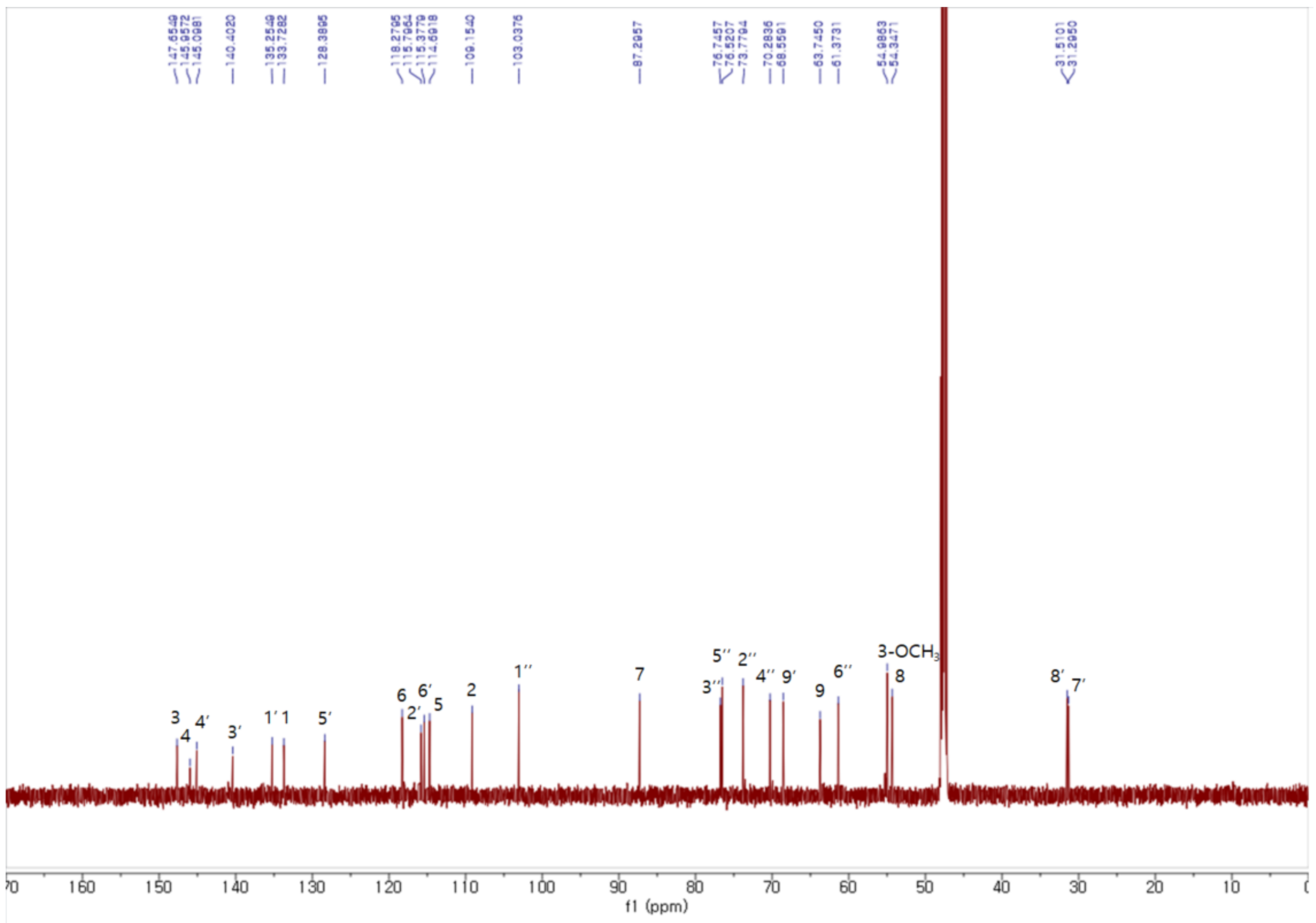

Figure S3 ^{13}C NMR data of **1** (CD_3OD, 175 MHz)

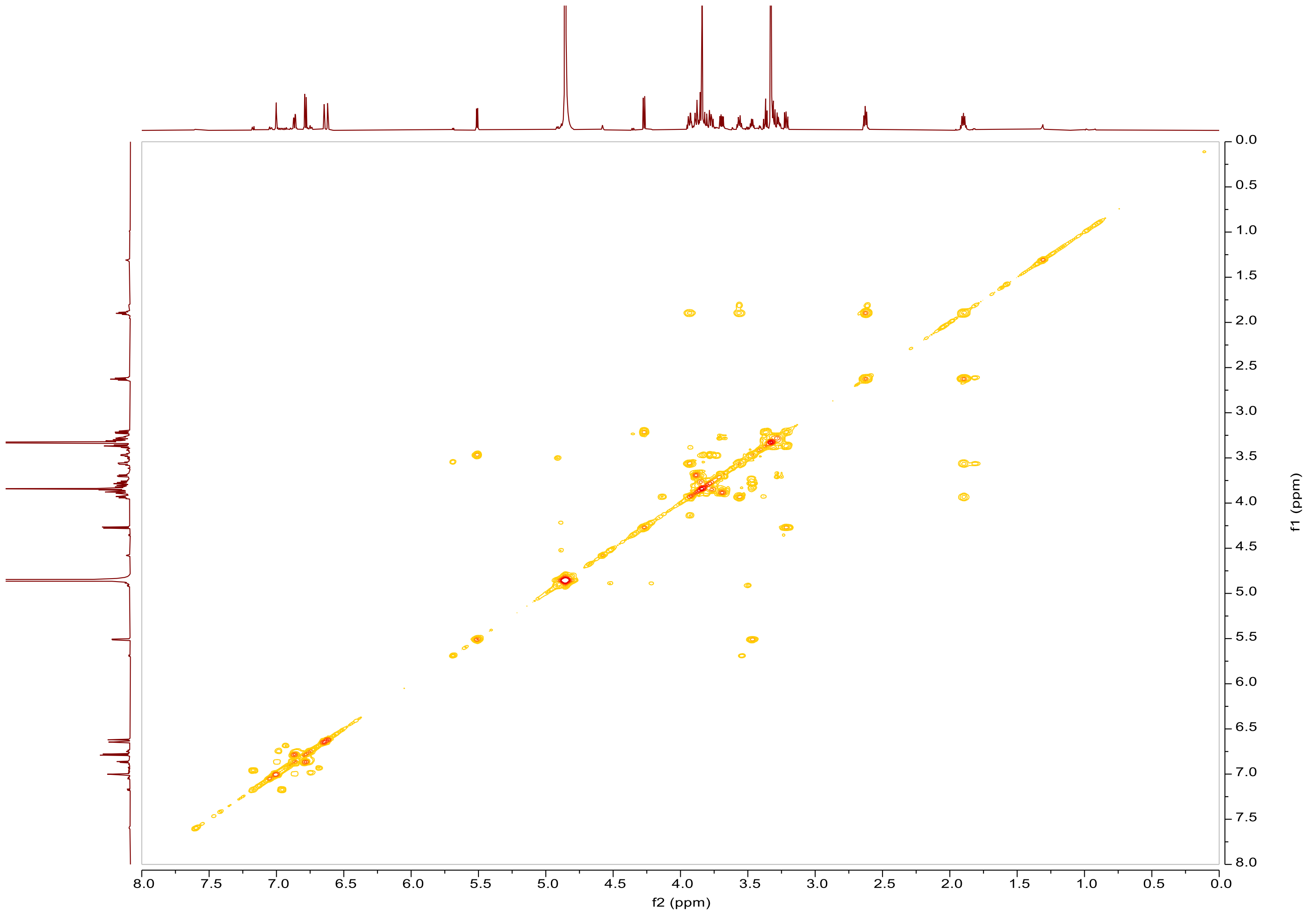

Figure S4 ^{1}H-^{1}H COSY data of **1** (CD_3OD)

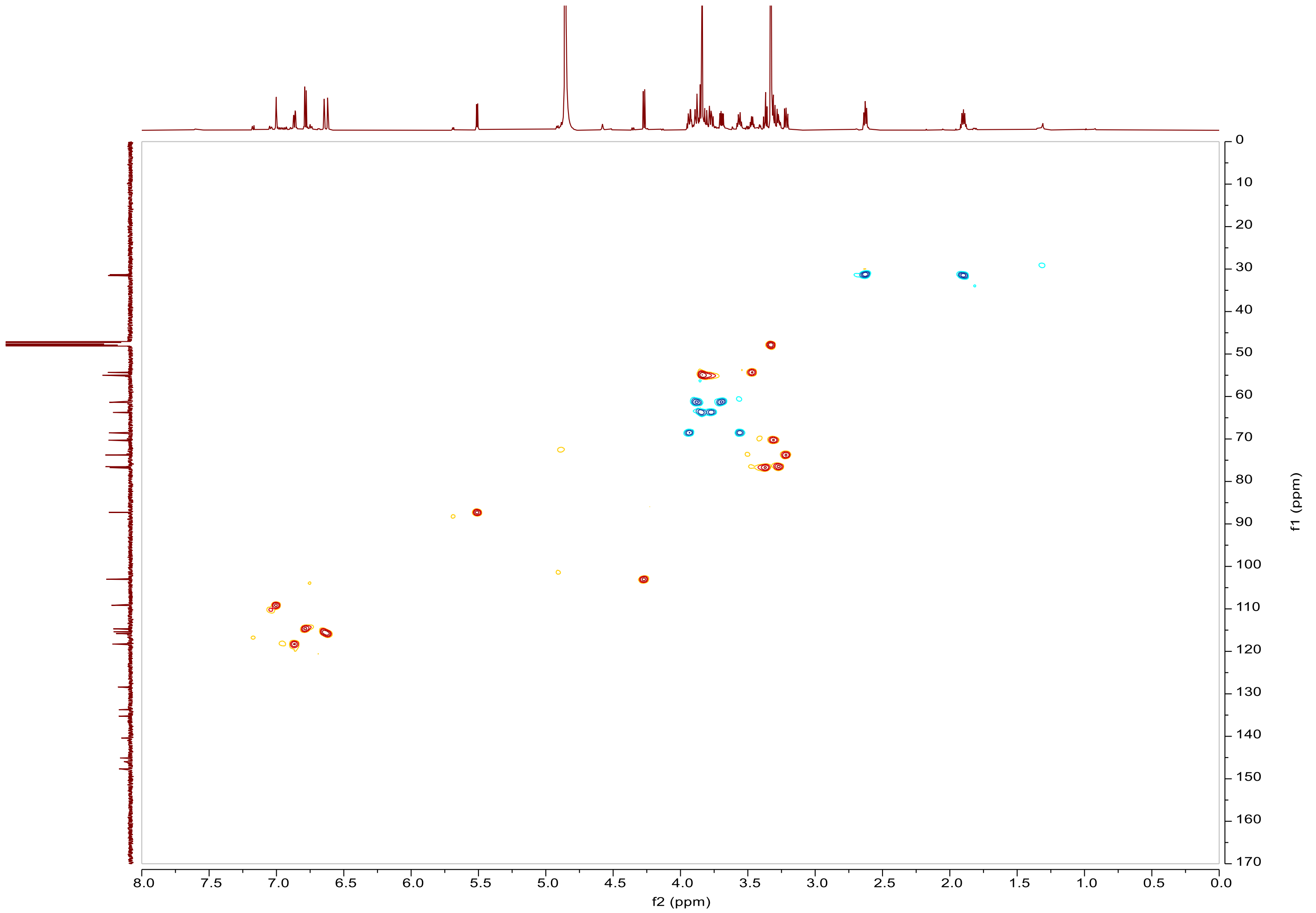

Figure S5 HSQC data of **1** (CD_3OD)

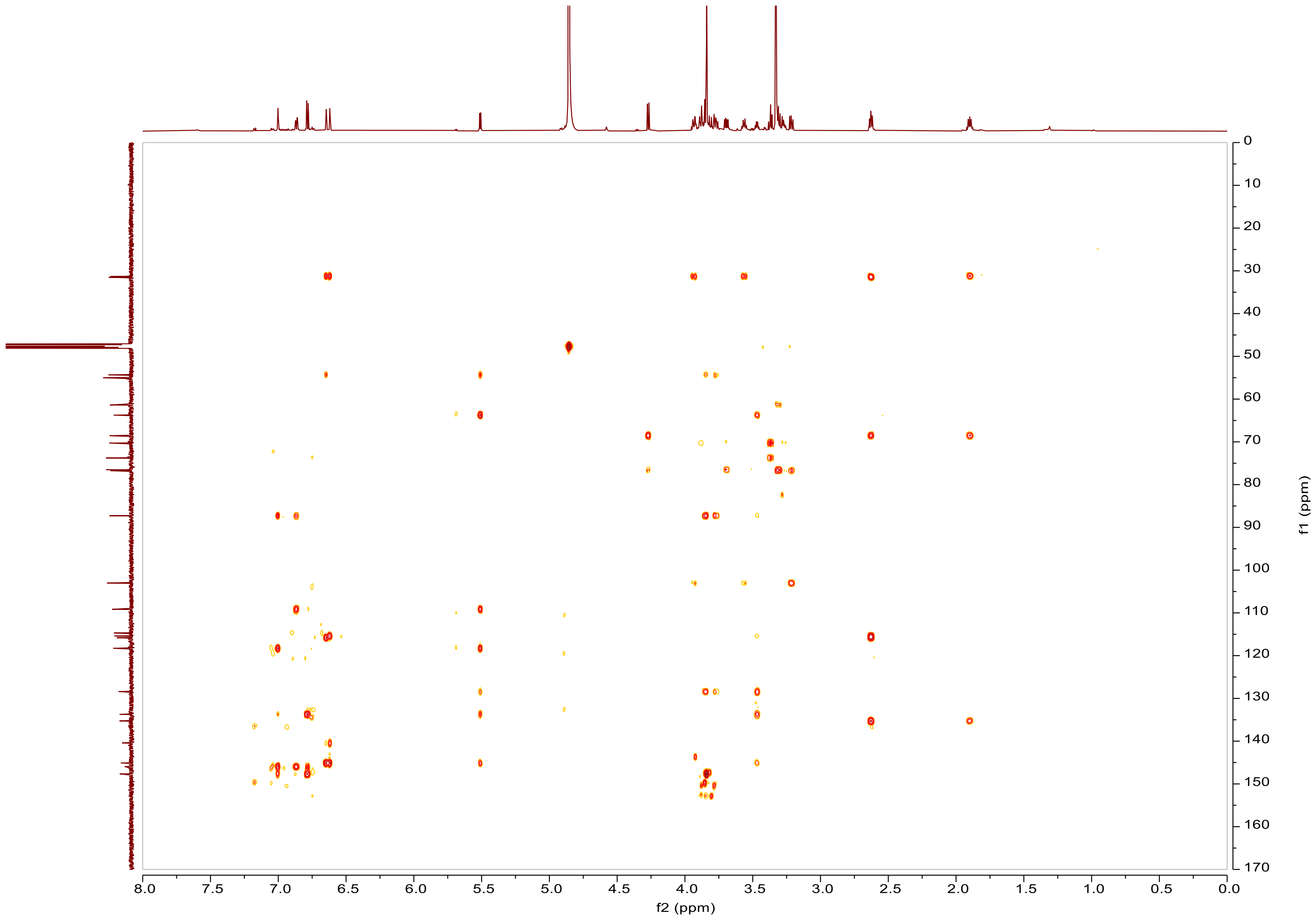

Figure S6 HMBC data of **1** (CD_3OD)

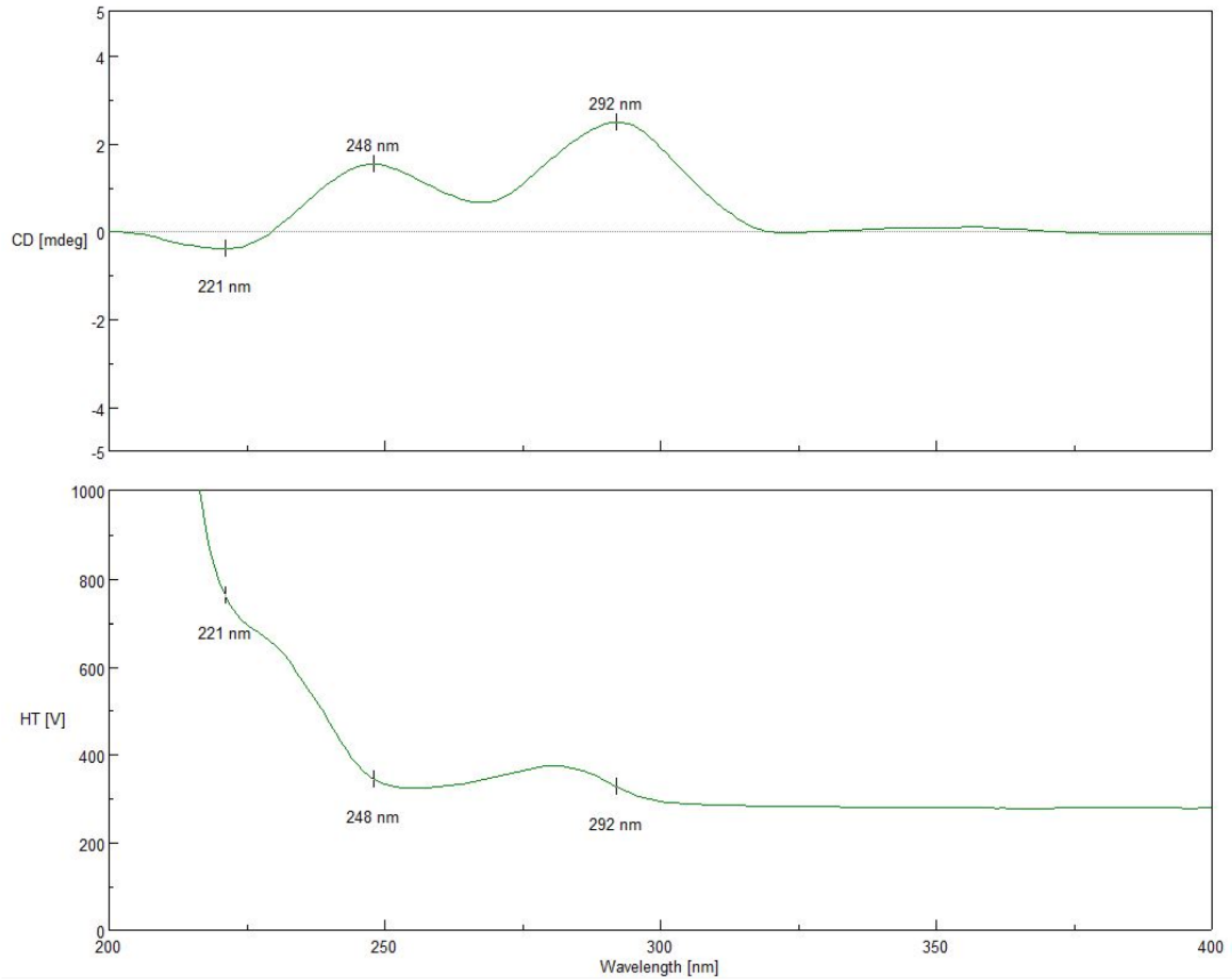

Figure S7 ECD data of **1**

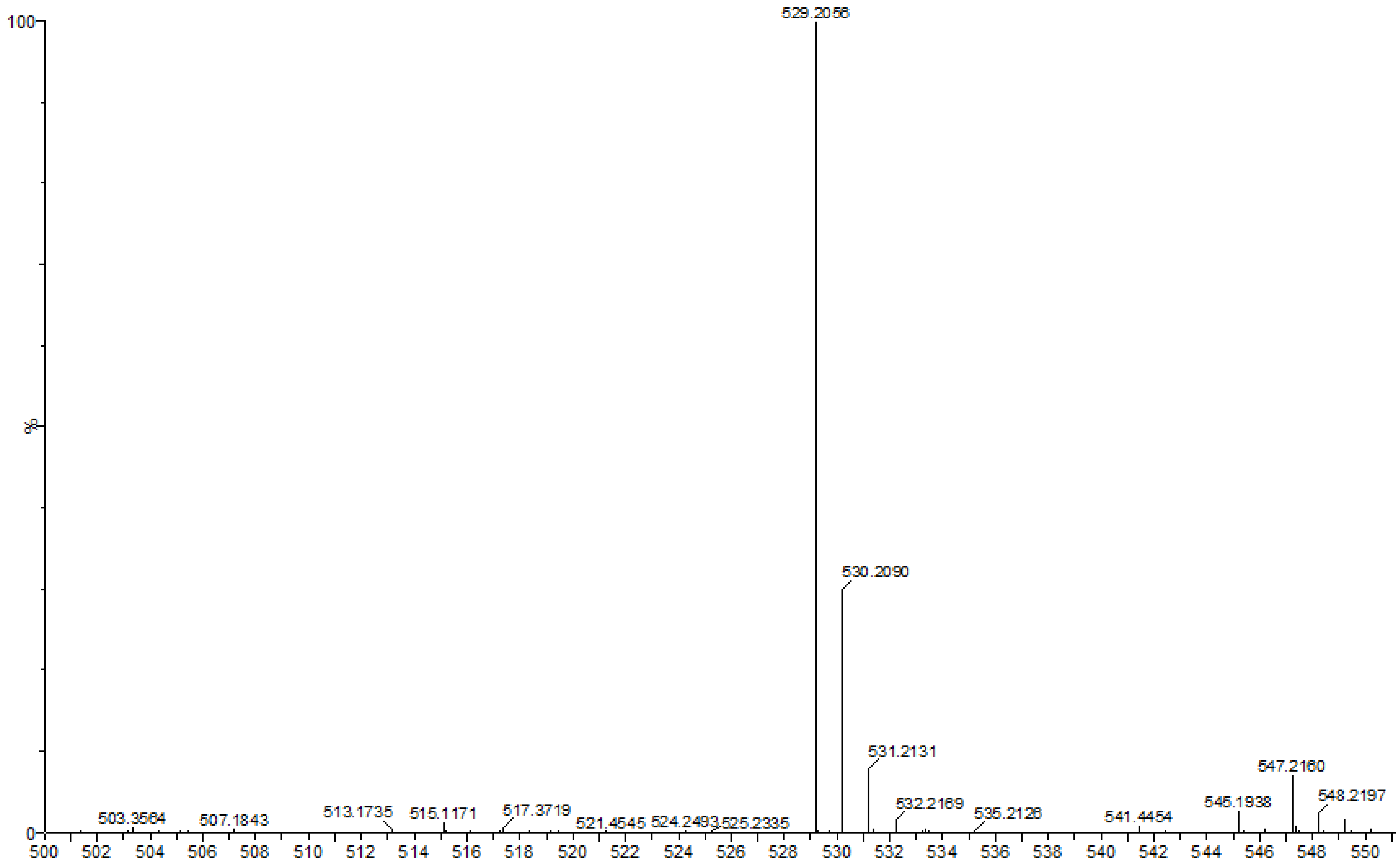

Figure S8 HRESIMS data of **2**

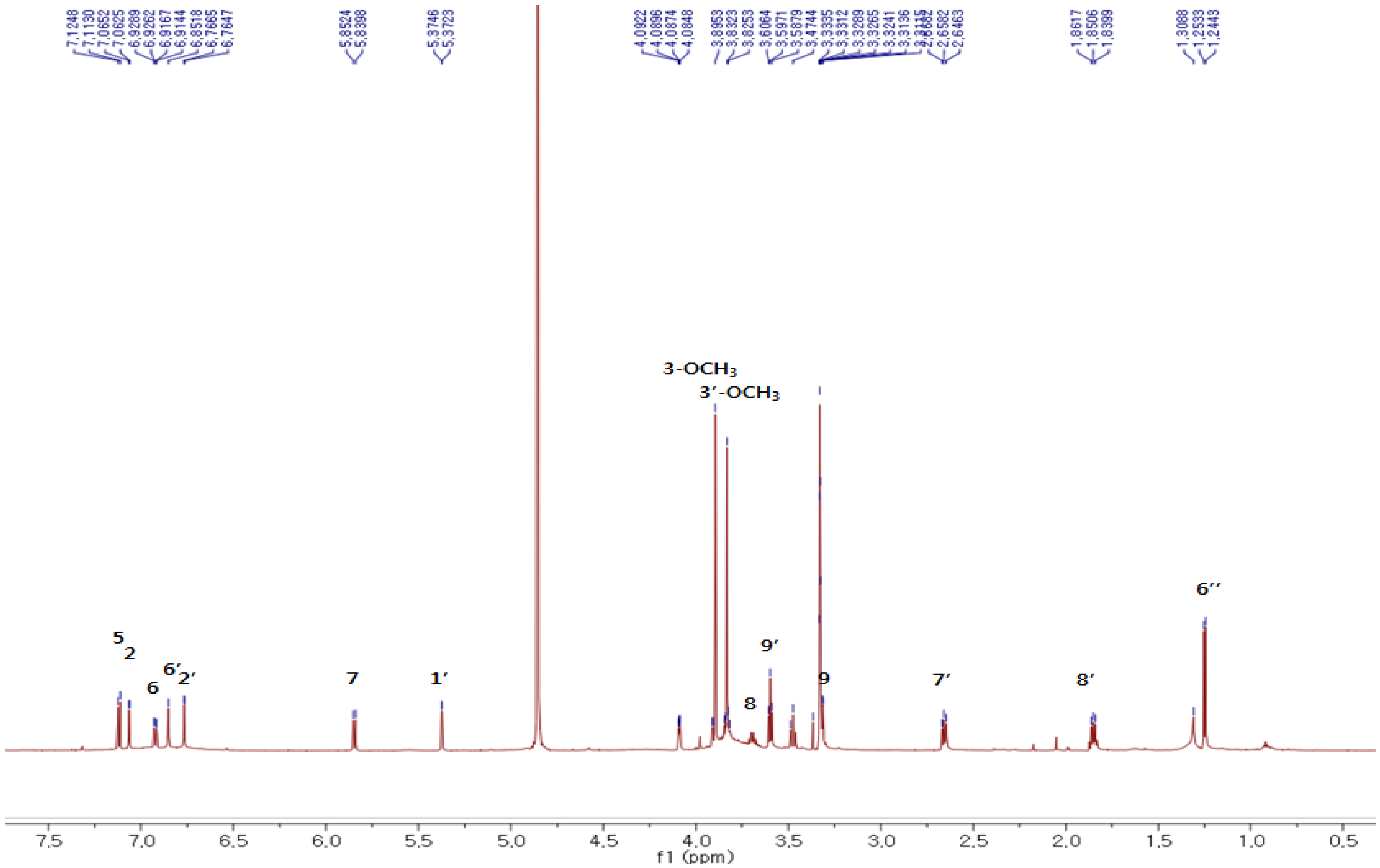

Figure S9 ^{1}H NMR data of **2** (CD_3OD, 500 MHz)

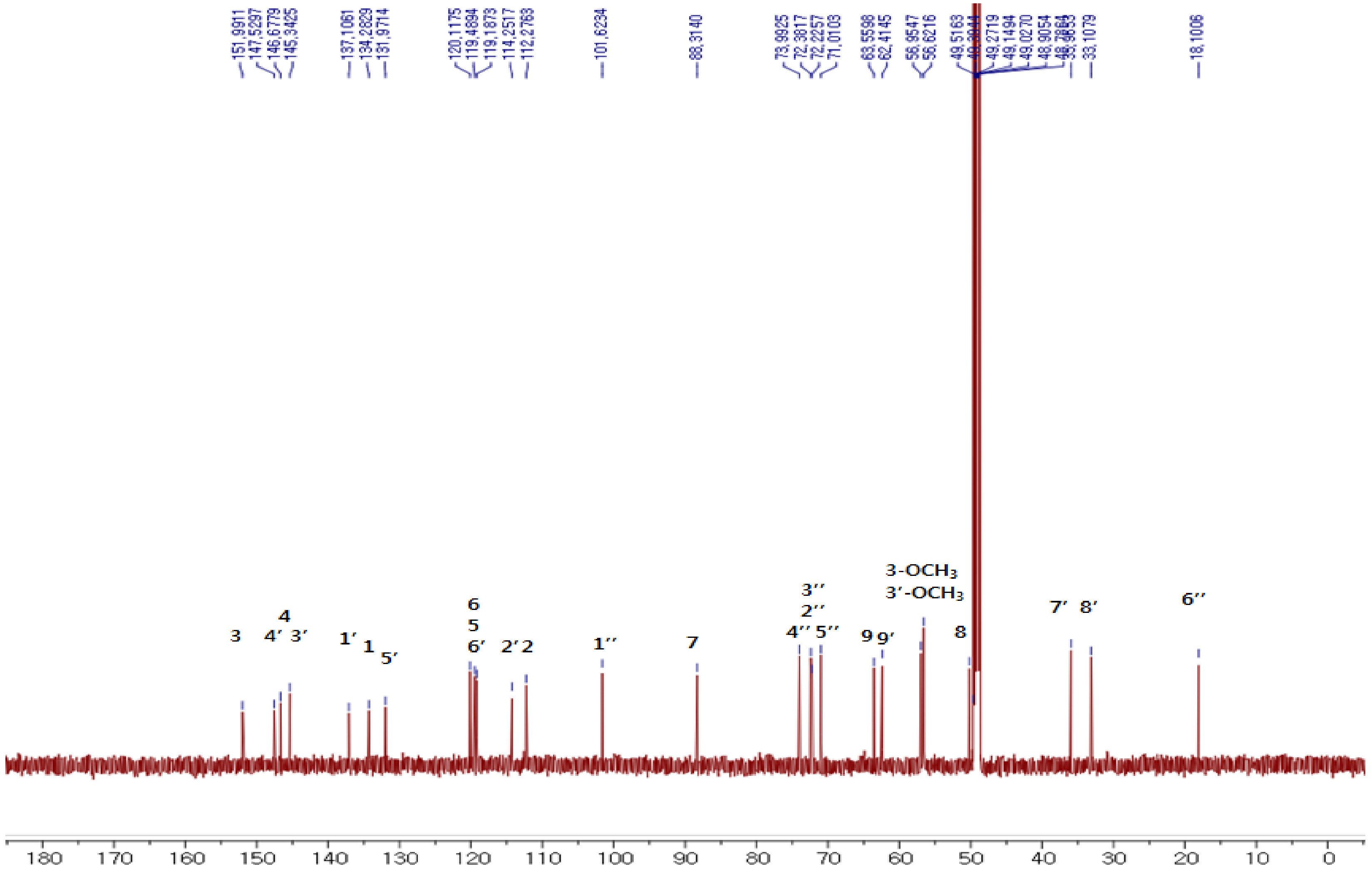

Figure S10 ^{13}C NMR data of **2** (CD_3OD, 125 MHz)

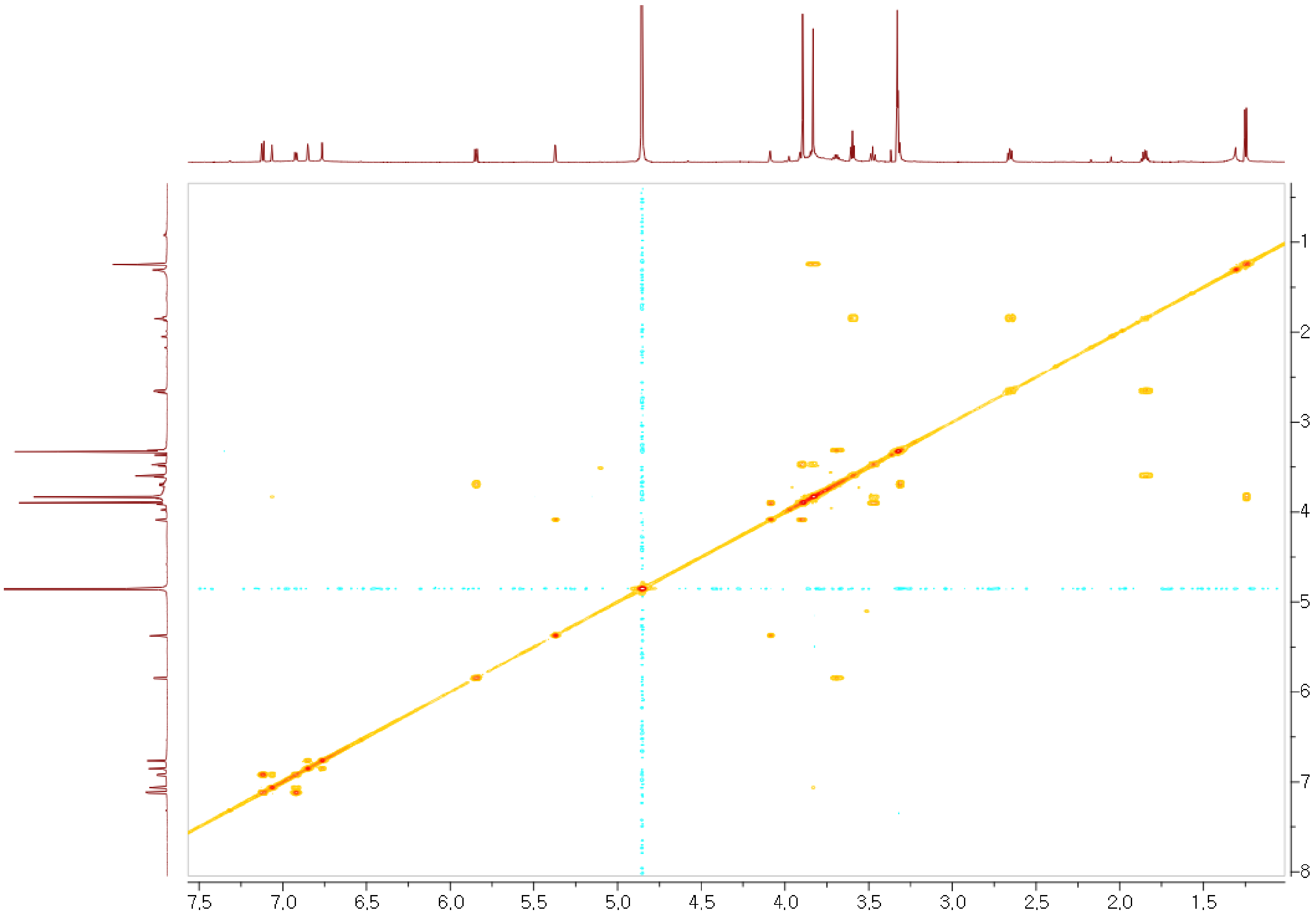

Figure S11 ^{1}H-^{1}H COSY data of **2** (CD_3OD)

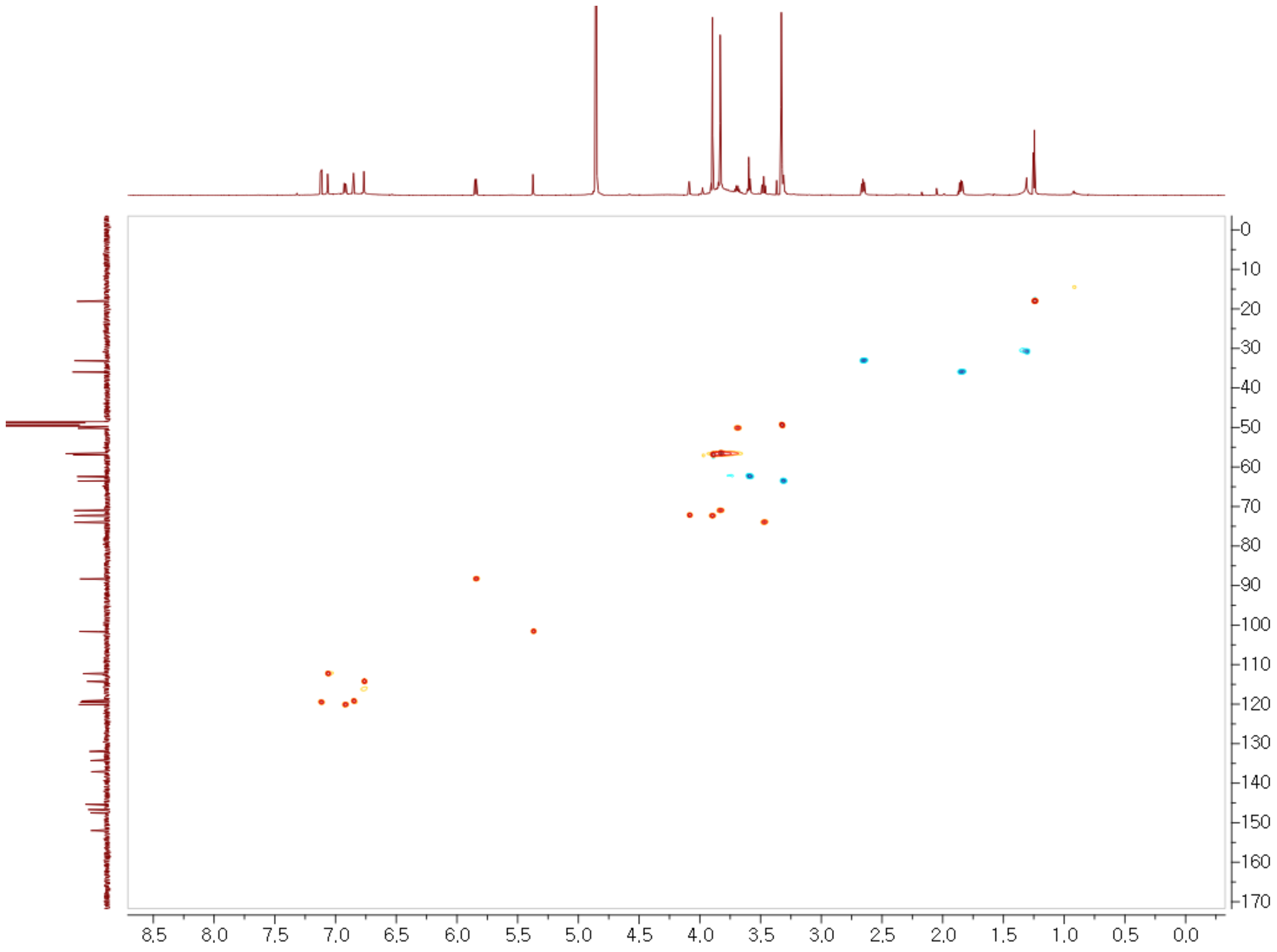

Figure S12 HSQC data of **2** (CD_3OD)

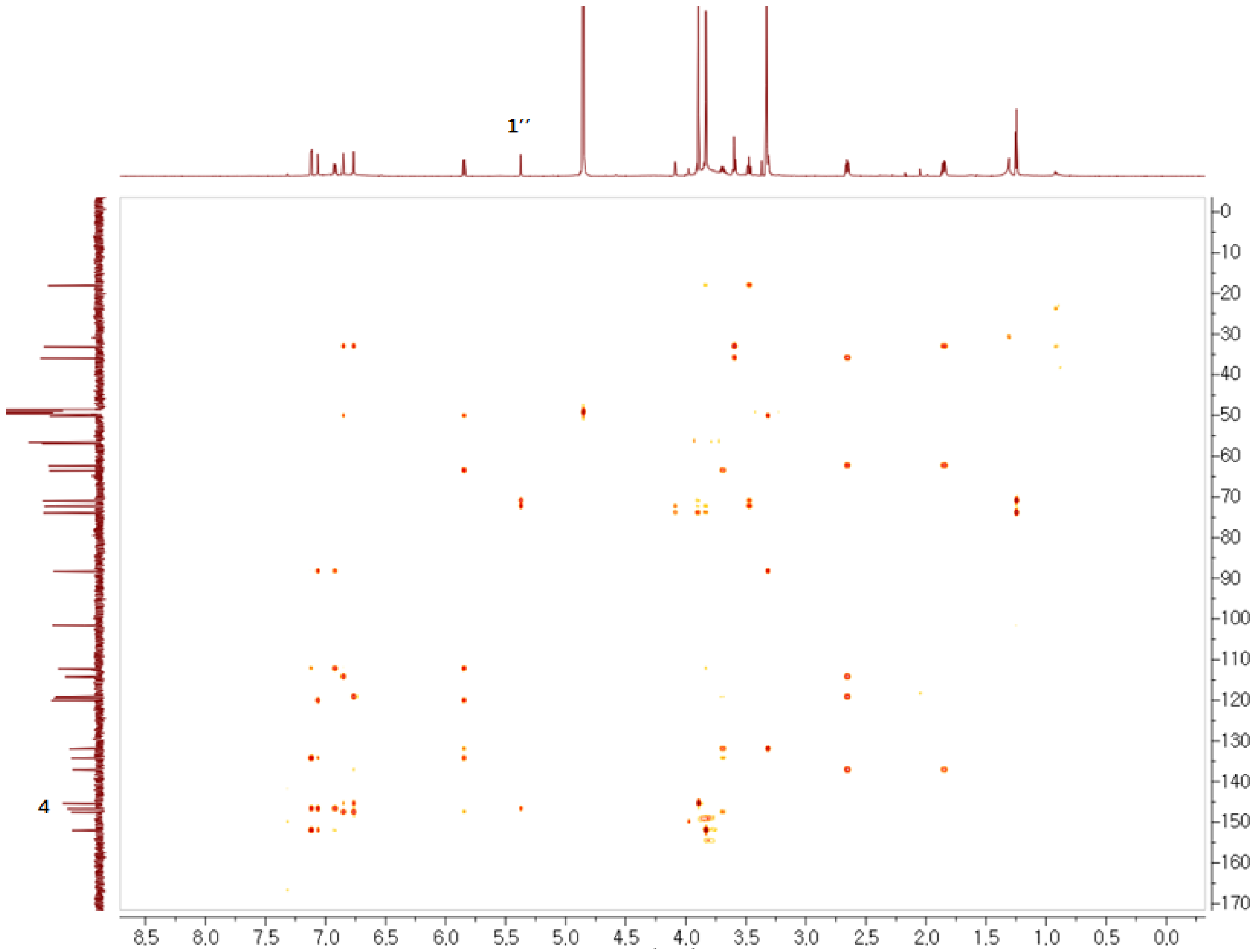

Figure S13 HMBC data of **2** (CD_3OD)

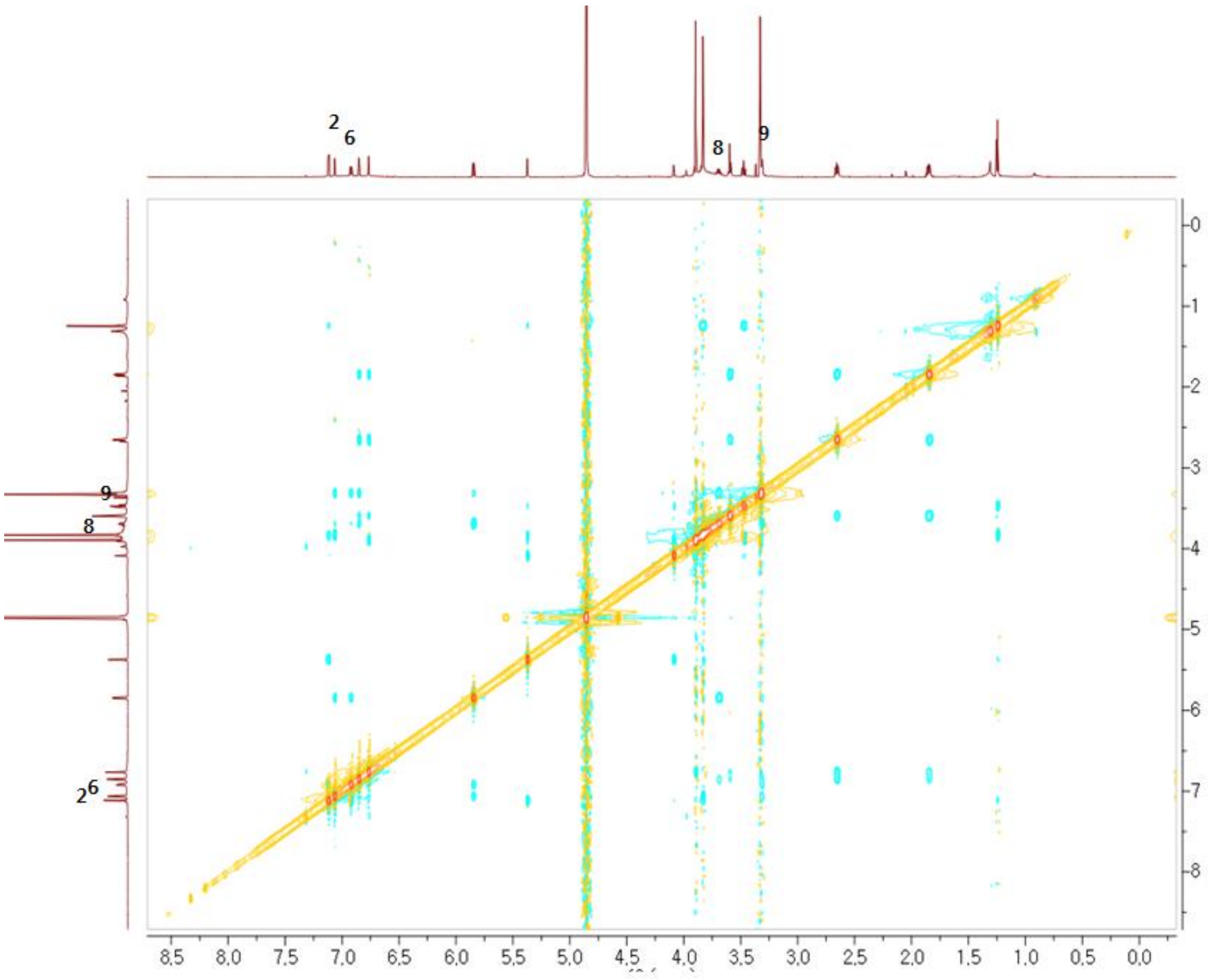

Figure S14 NOESY data of **2** (CD_3OD)

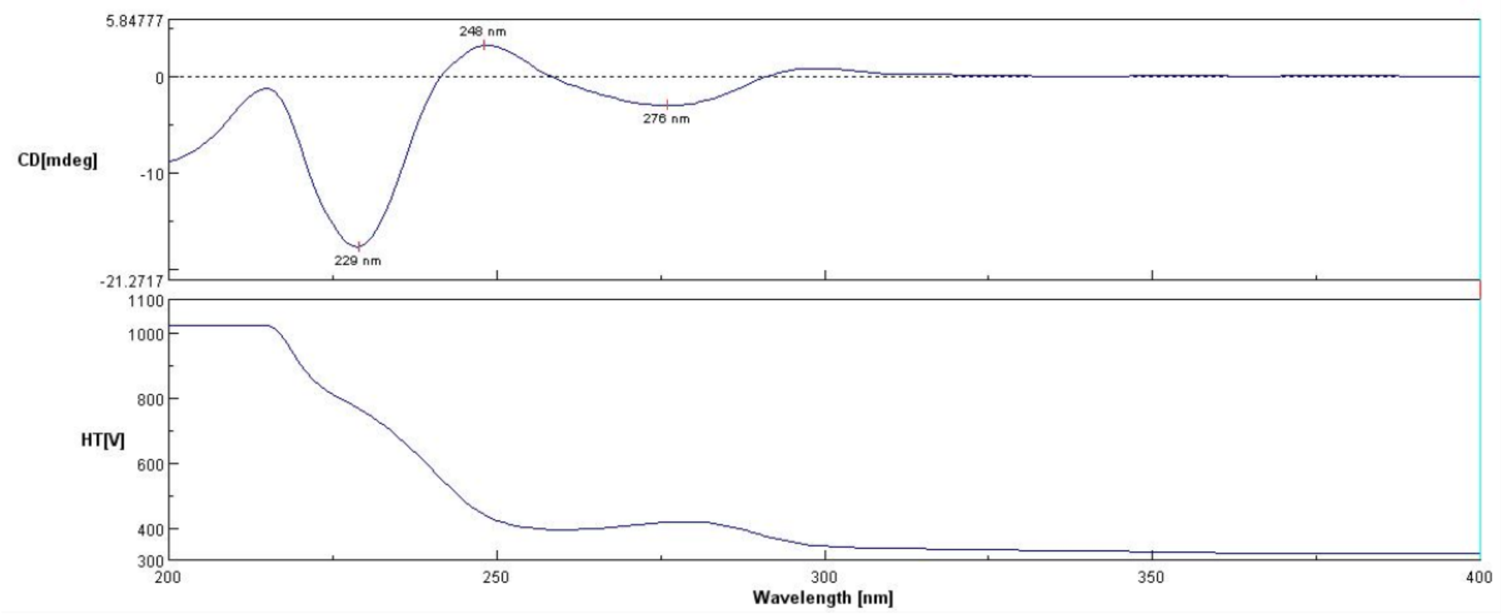

Figure S15 ECD data of **2**

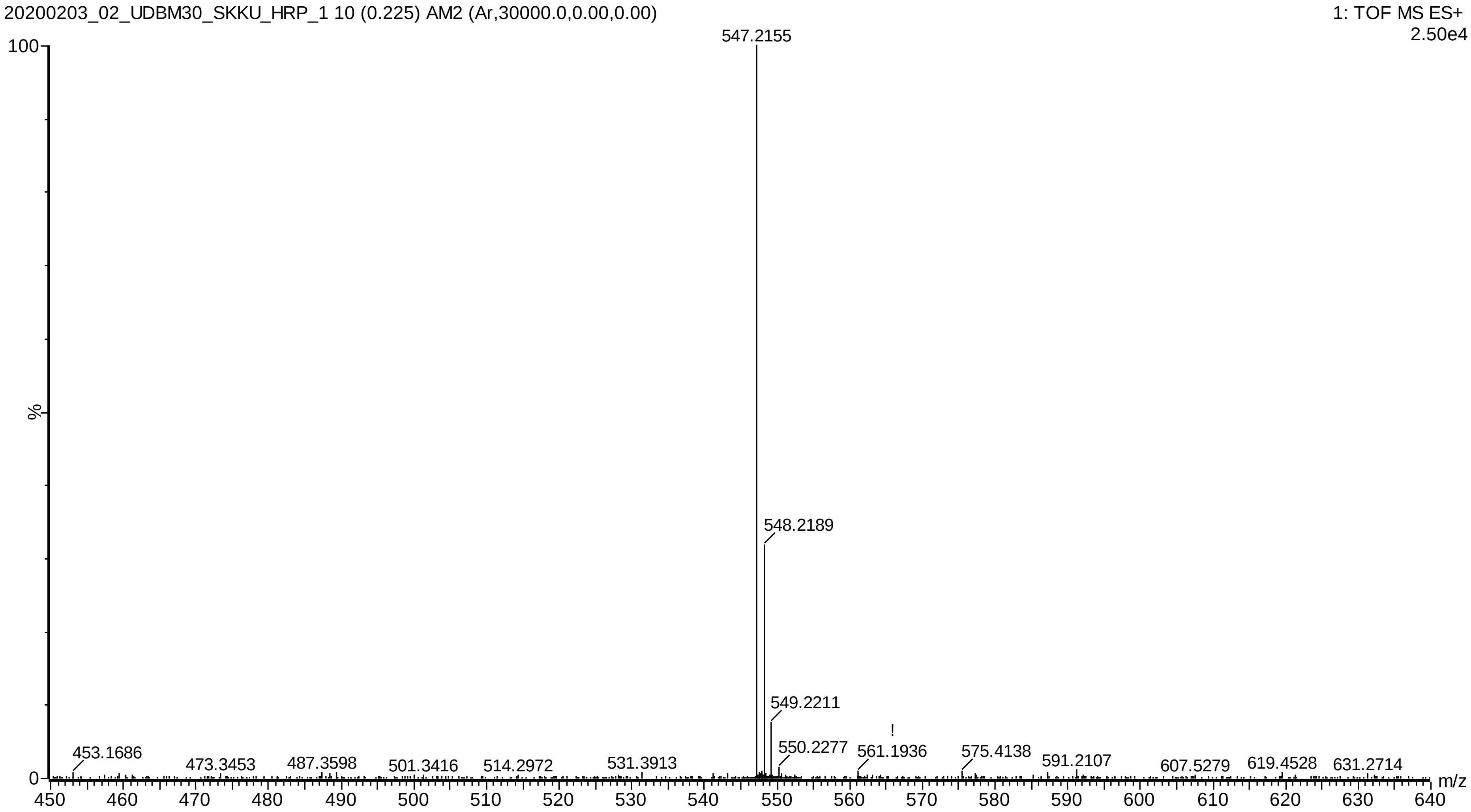

Figure S16 HRESIMS data of **3**

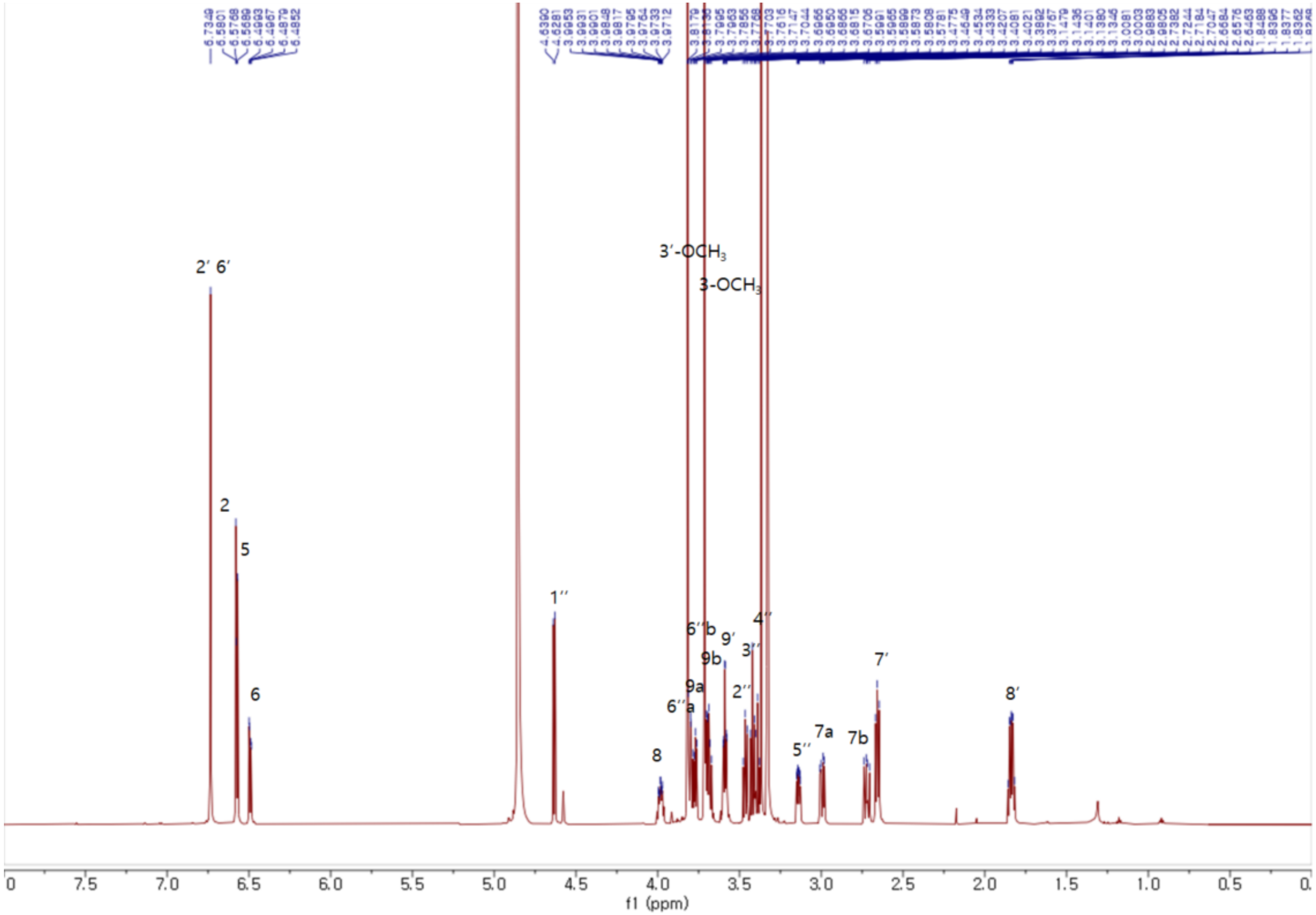

Figure S17 ^{1}H NMR data of **3** (CD$_3$OD, 700 MHz)

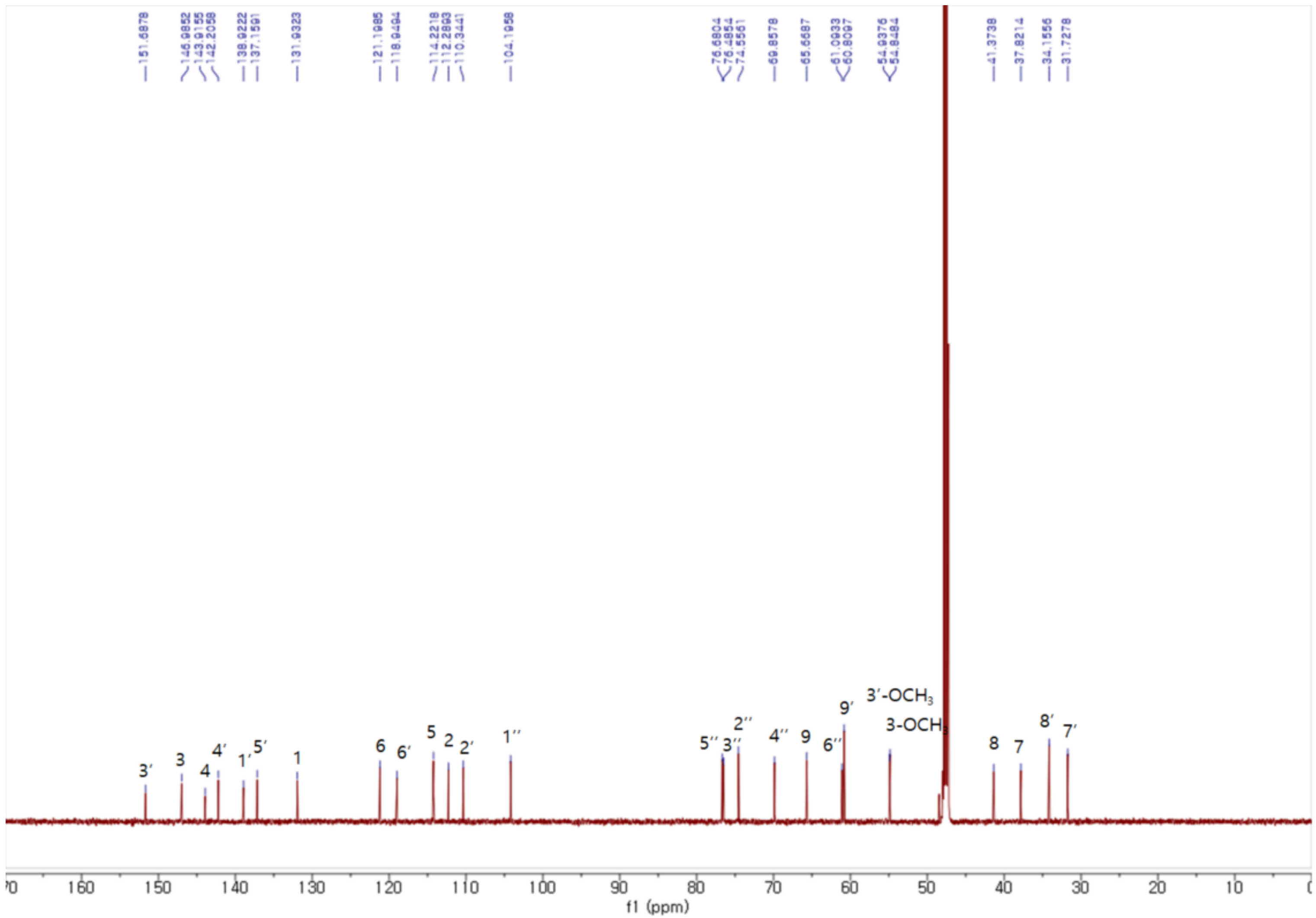

Figure S18 ^{13}C NMR data of **3** (CD_3OD, 175 MHz)

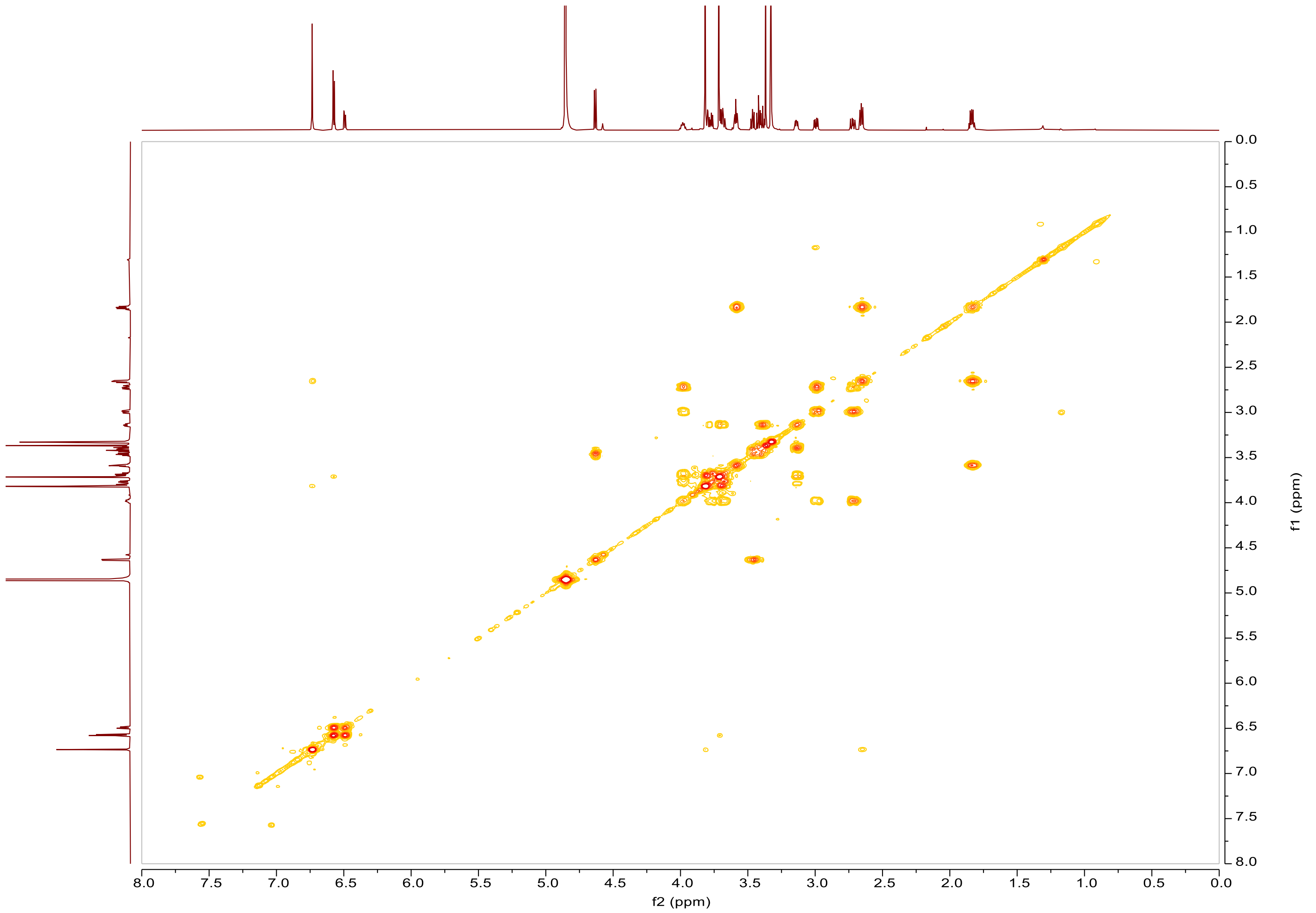

Figure S19 ^{1}H-^{1}H COSY data of **3** (CD_3OD)

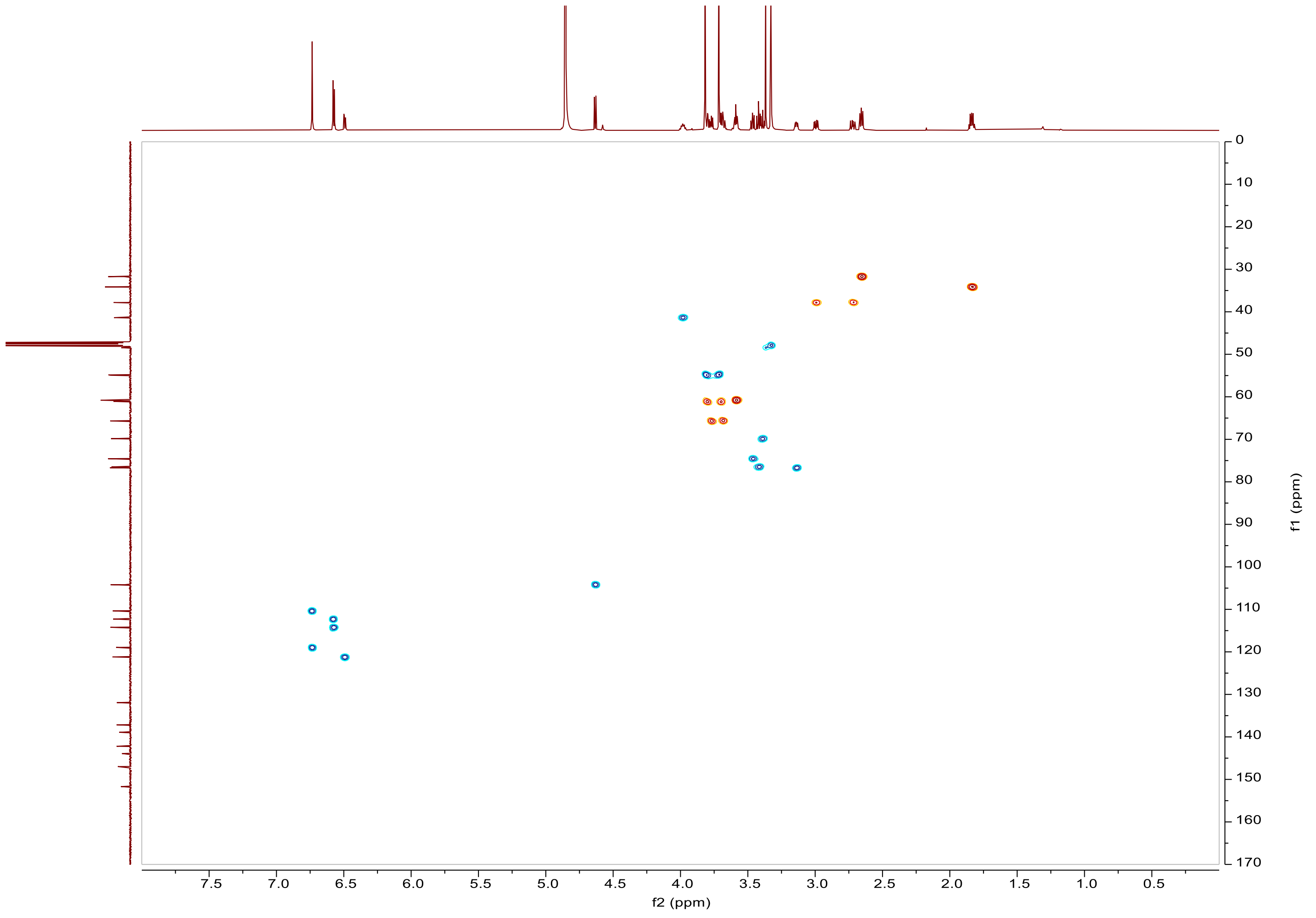

Figure S20 HSQC data of **3** (CD_3OD)

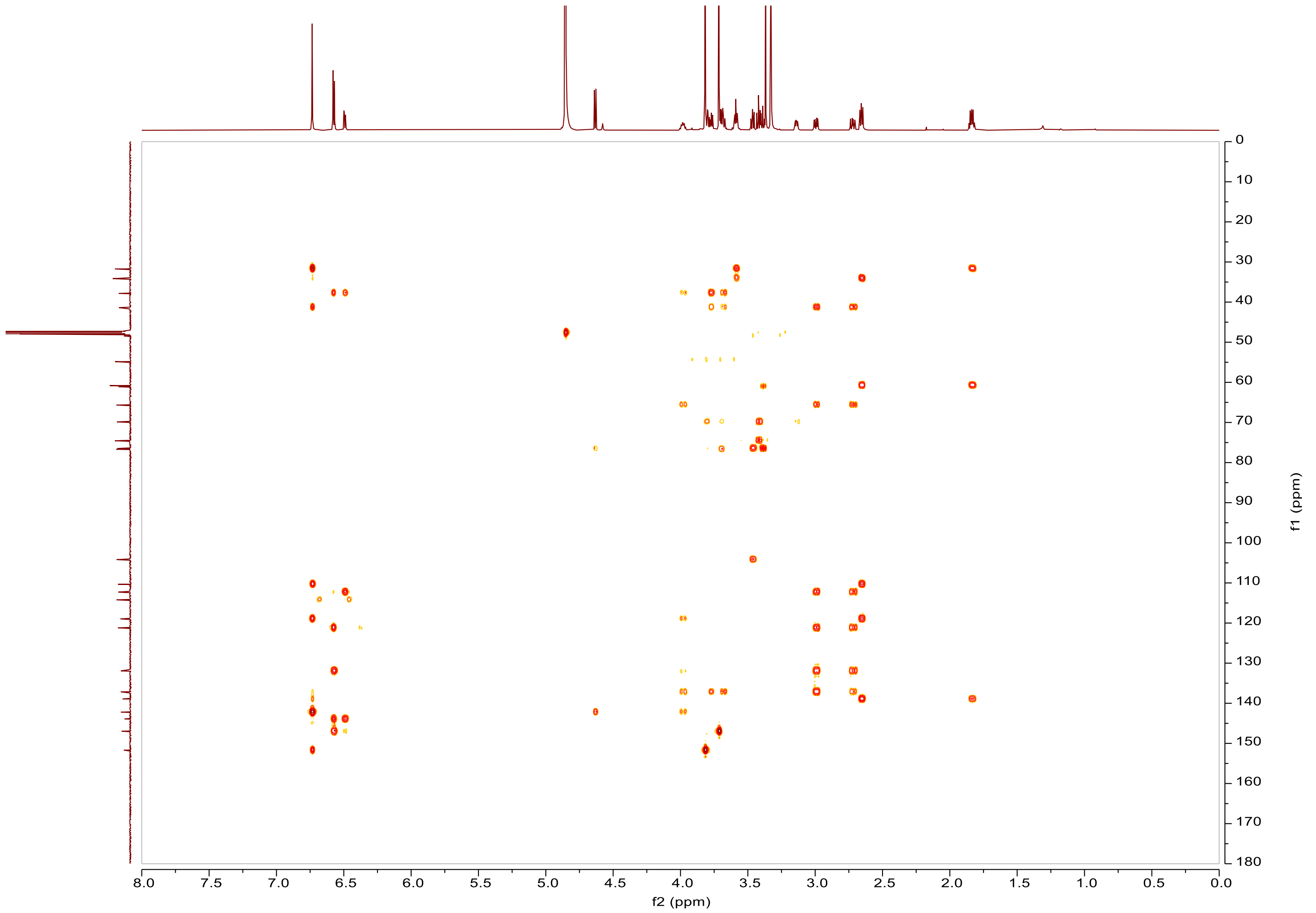

Figure S21 HMBC data of **3** (CD_3OD)

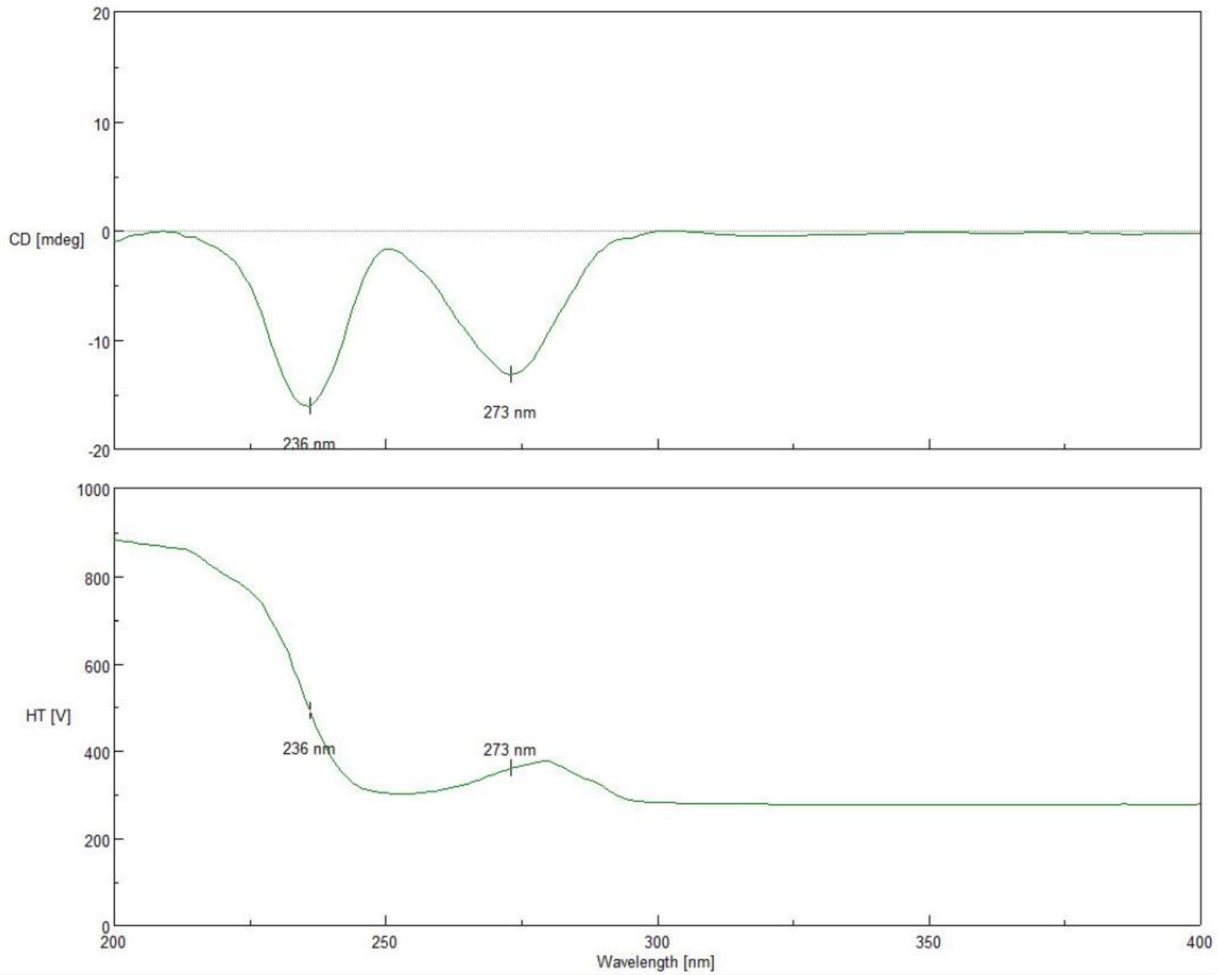

Figure S22 ECD data of **3**

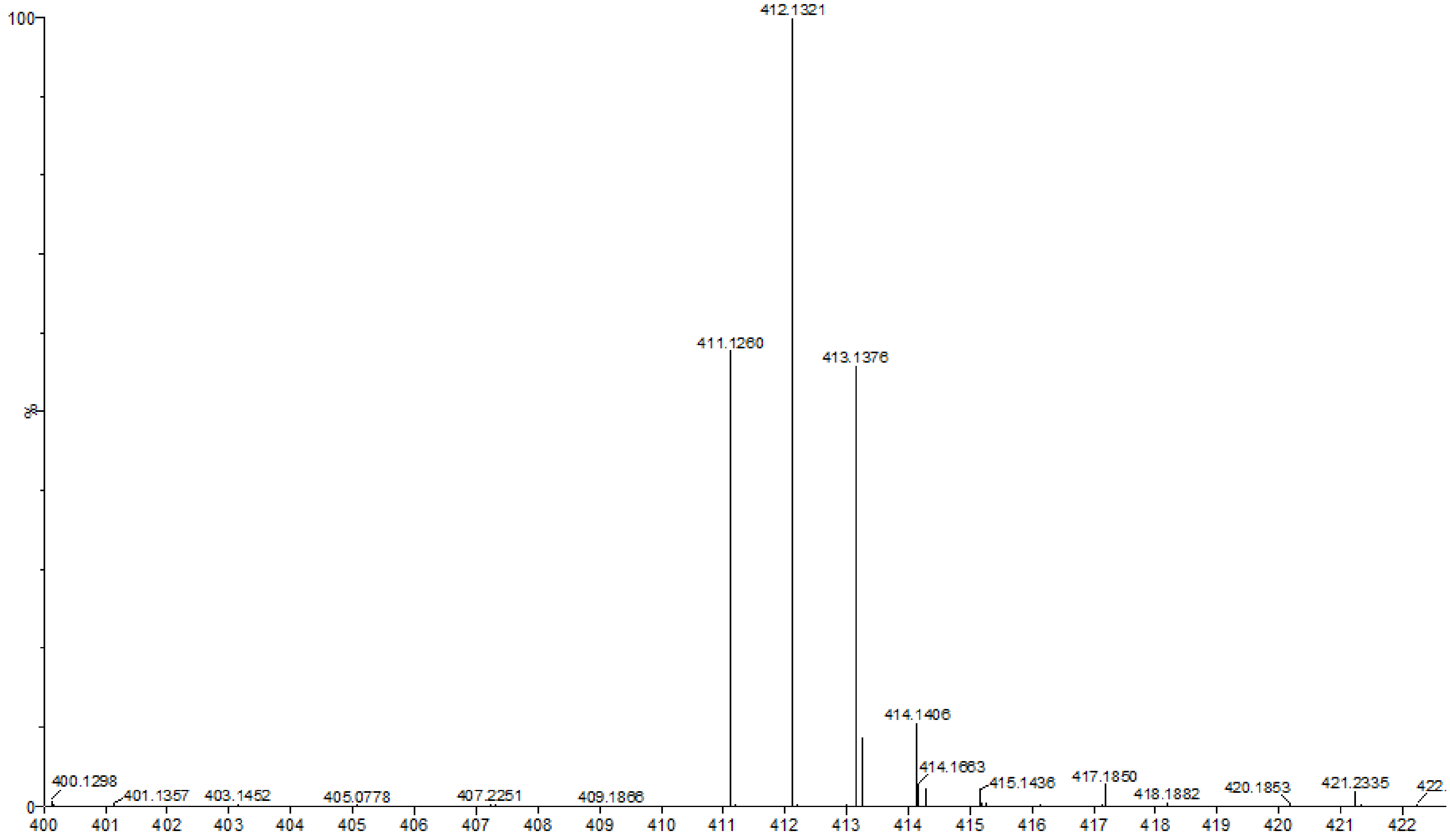

Figure S23 HRESIMS data of **15**

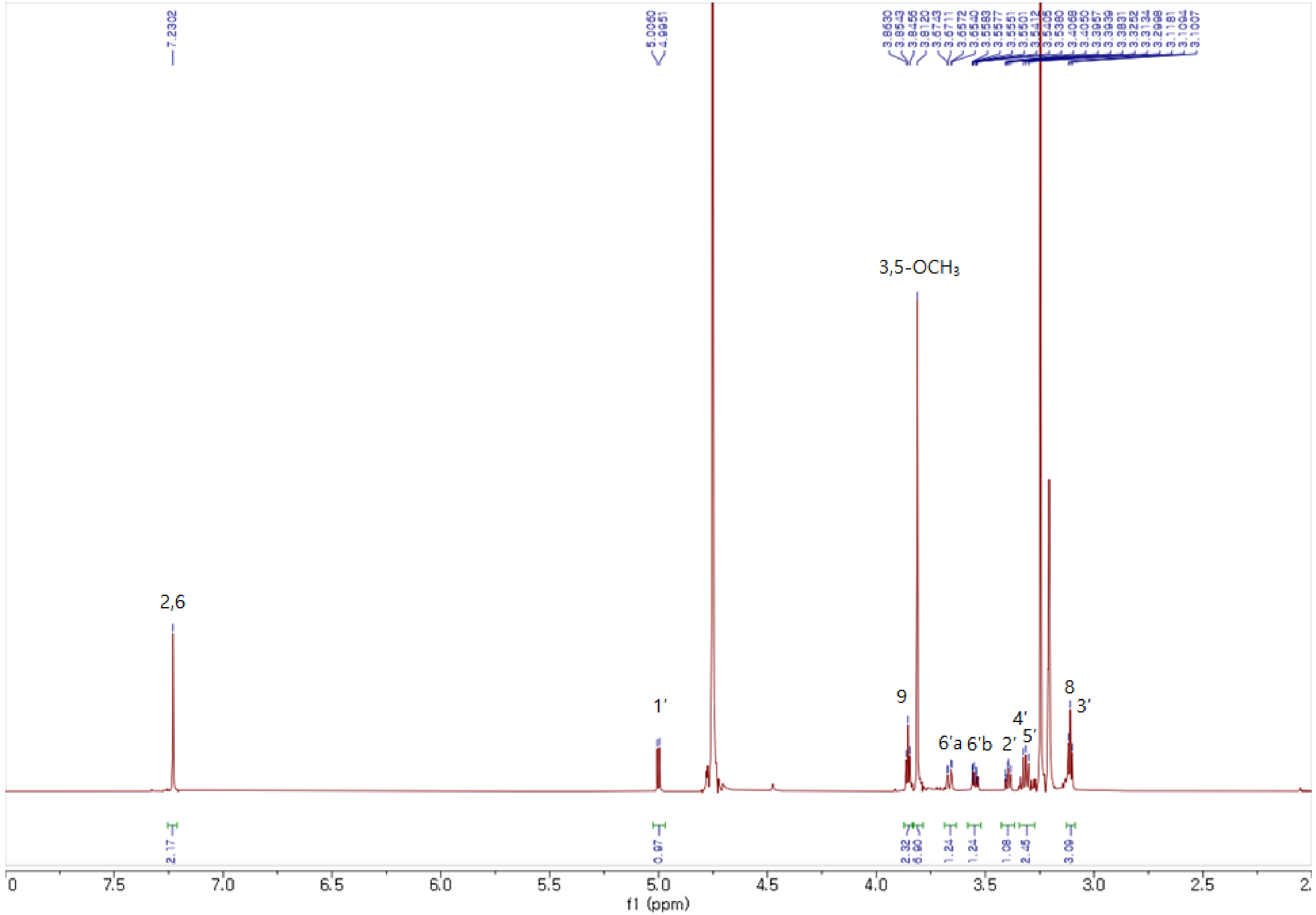

Figure S24 ^{1}H NMR data of **15** (CD_3OD, 700 MHz)

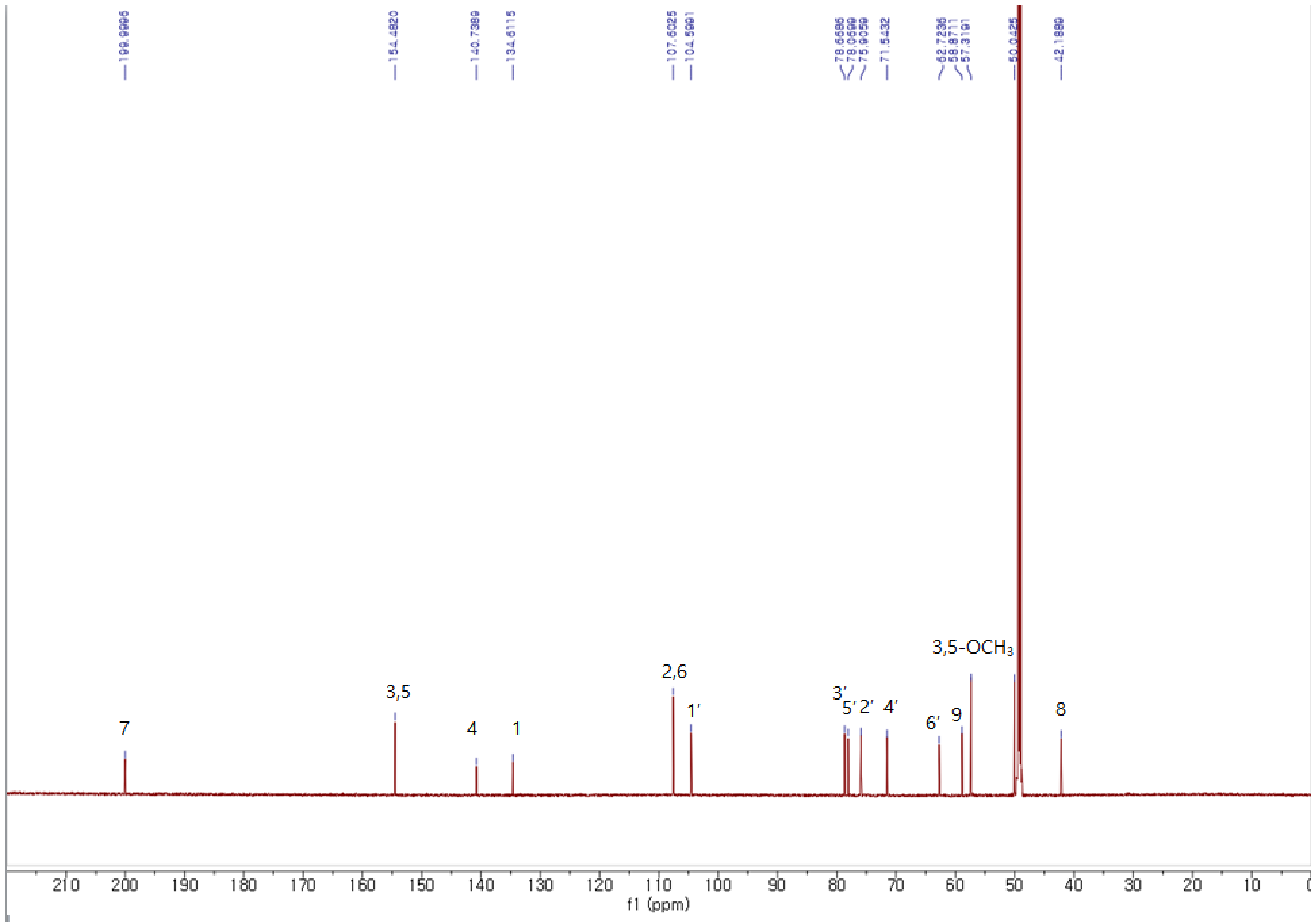

Figure S25 ^{13}C NMR data of **15** (CD_3OD, 175 MHz)

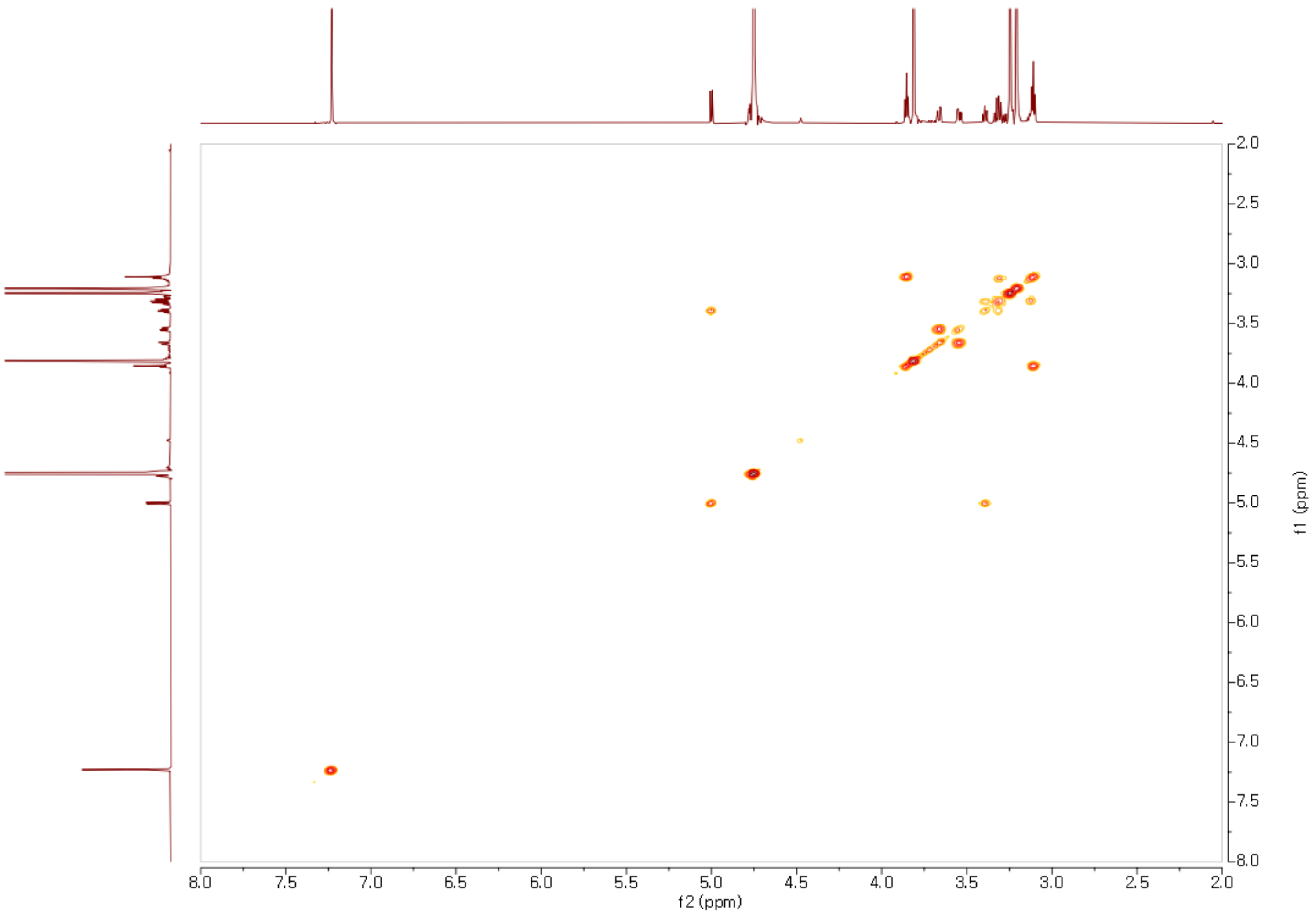

Figure S26 ^{1}H-^{1}H COSY data of **15** (CD_3OD)

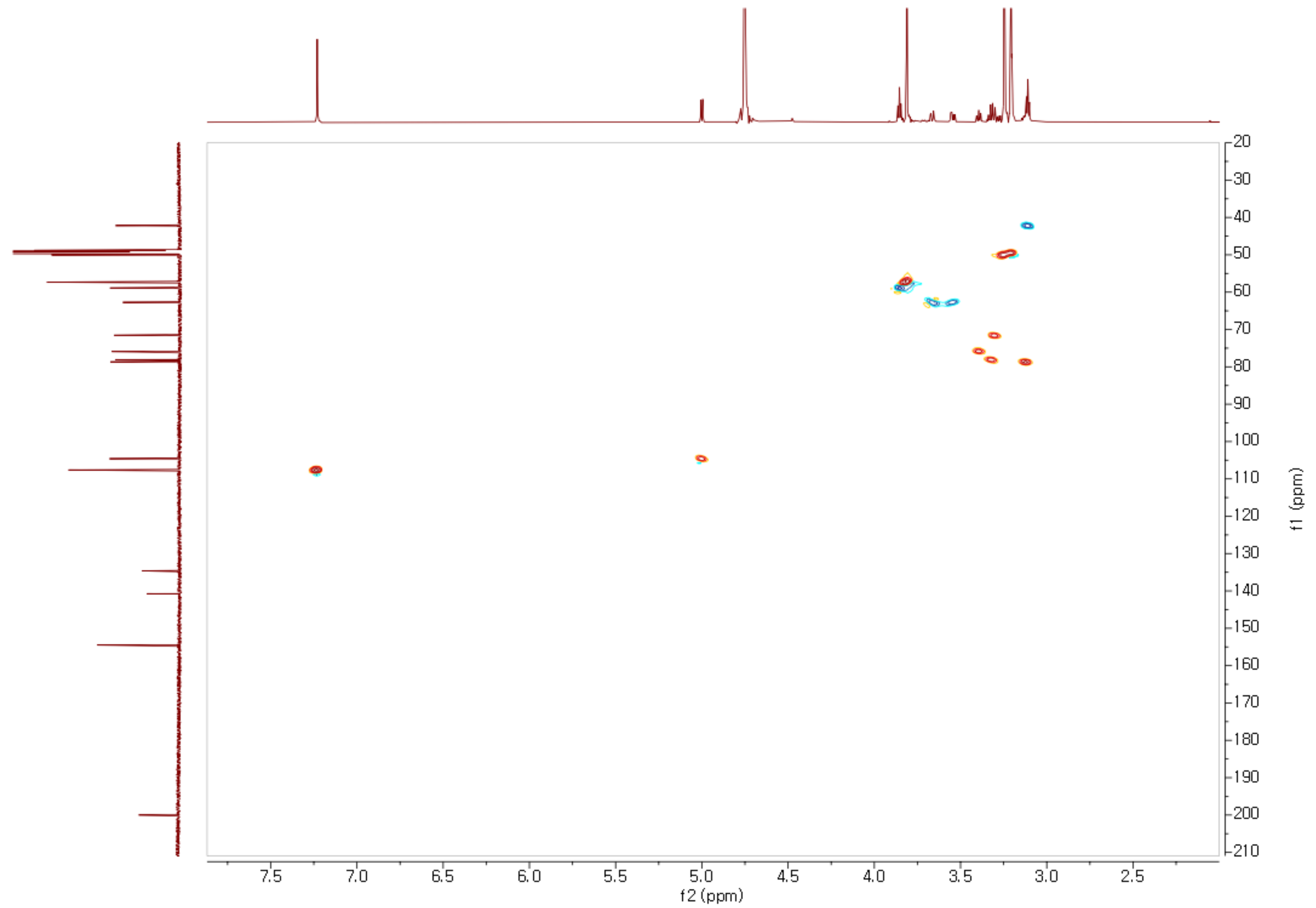

Figure S27 HSQC data of **15** (CD_3OD)

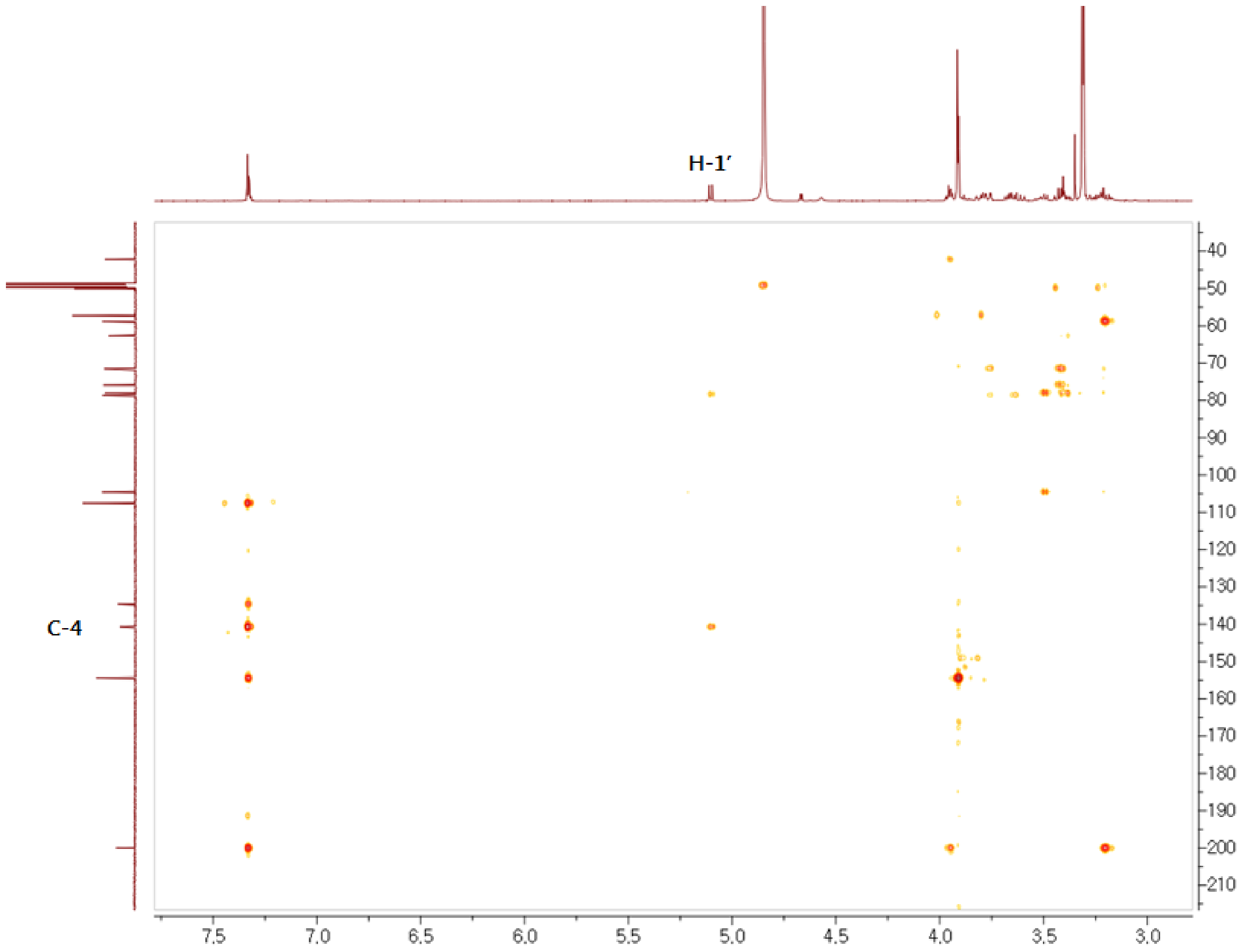

Figure S28 HMBC data of **15** (CD_3OD)

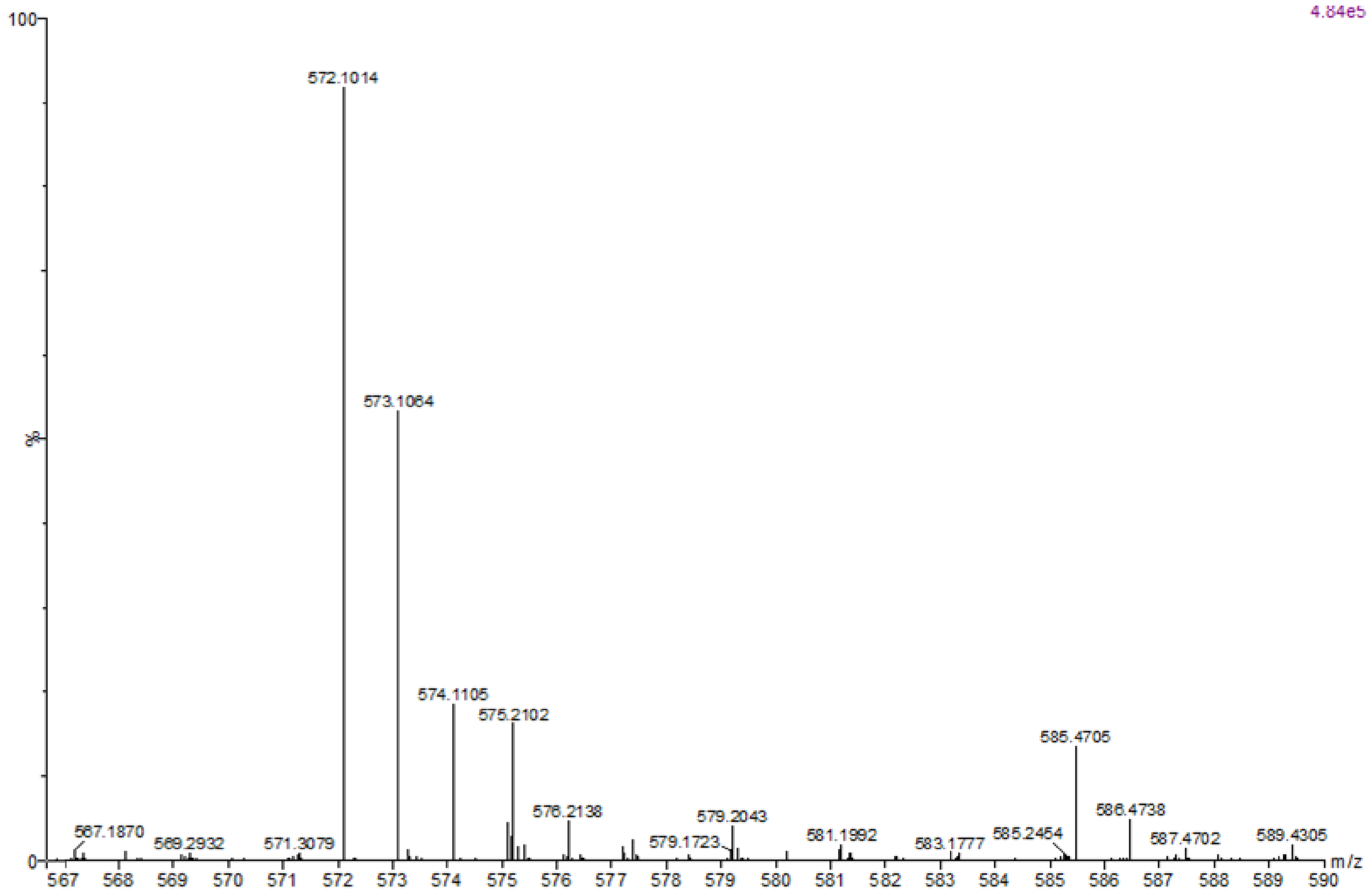

Figure S29 HRESIMS data of **16**

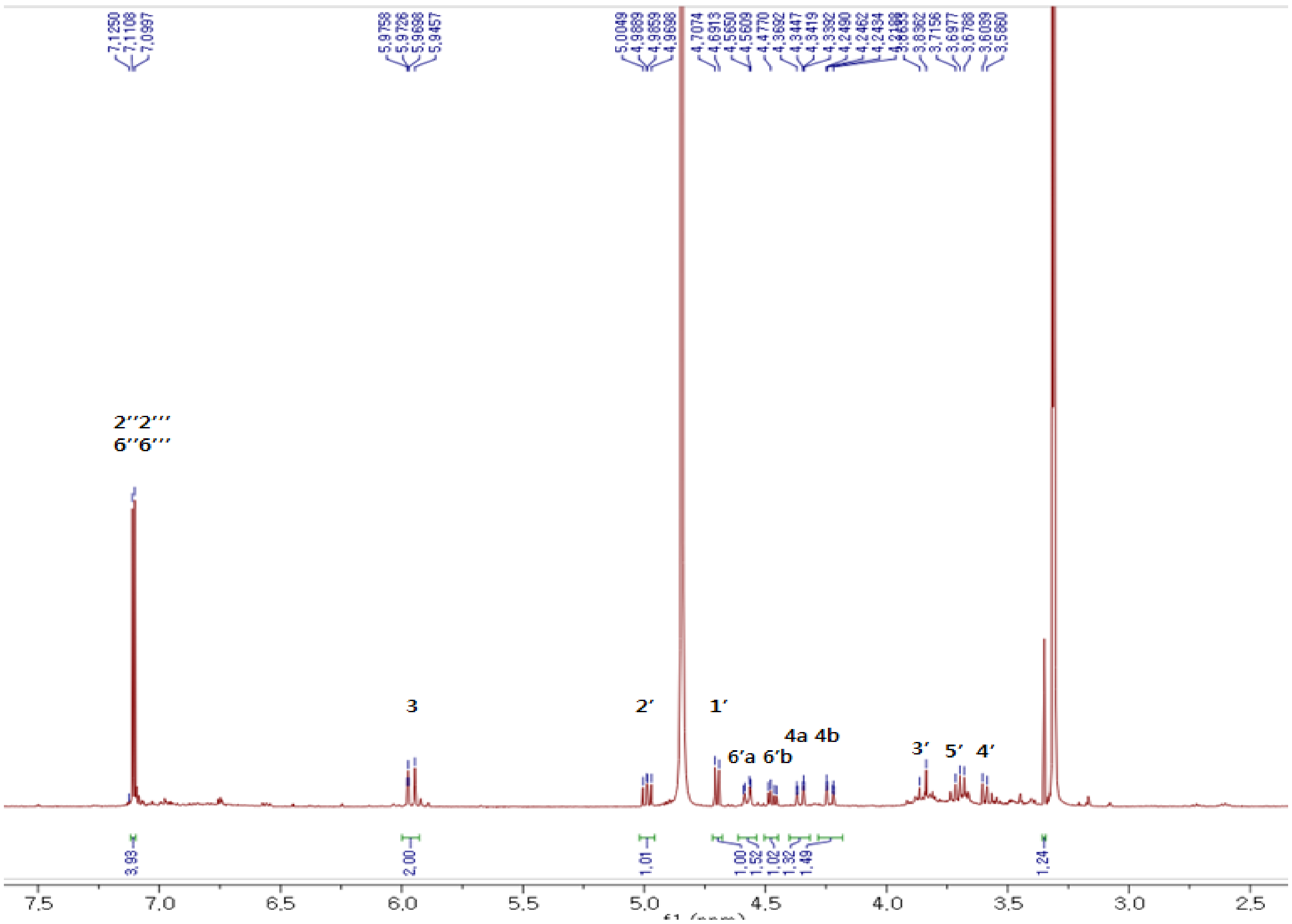

Figure S30 ^{1}H NMR data of **16** (CD_3OD, 500 MHz)

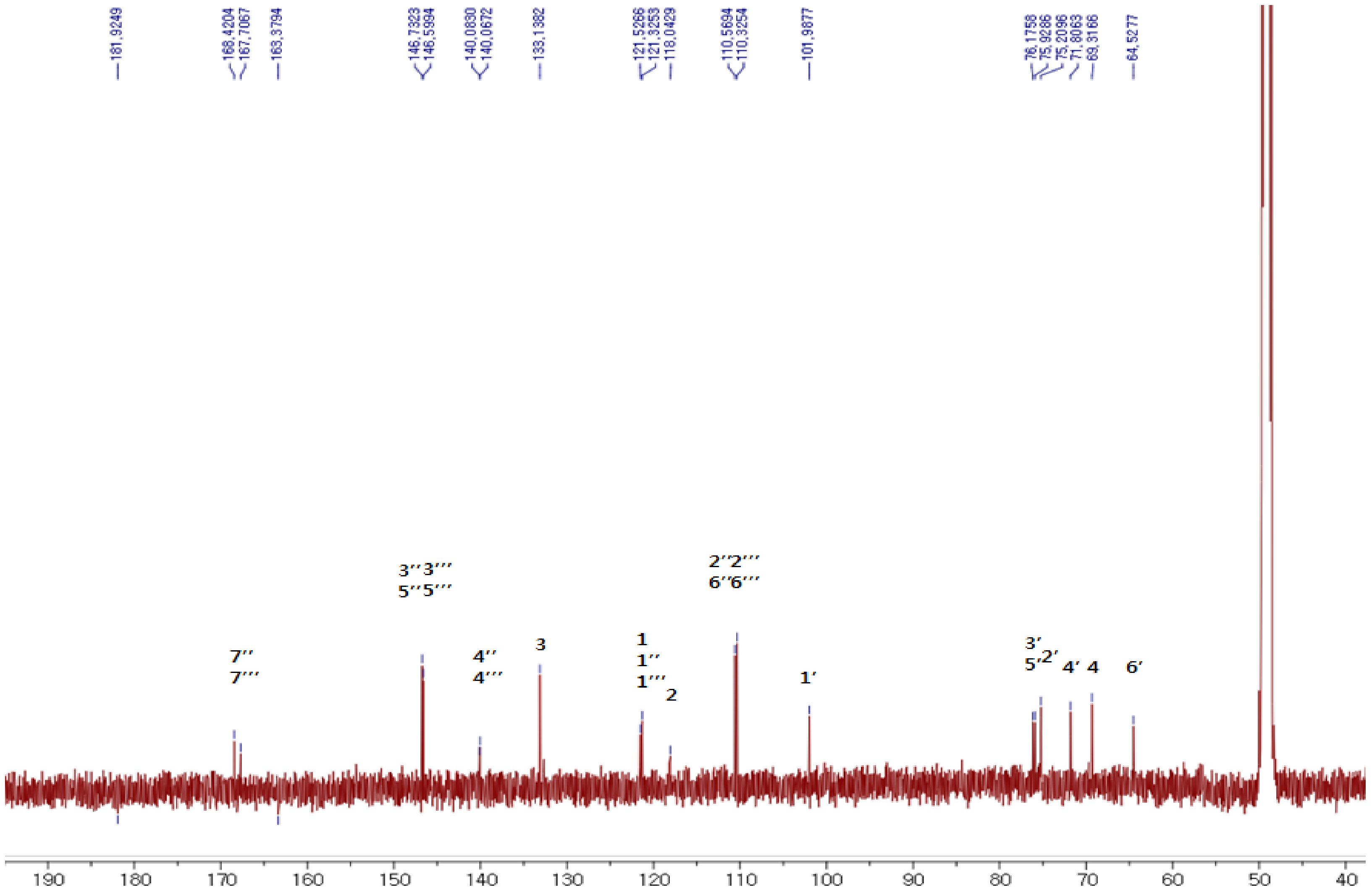

Figure S31 ^{13}C NMR data of **16** (CD_3OD, 125 MHz)

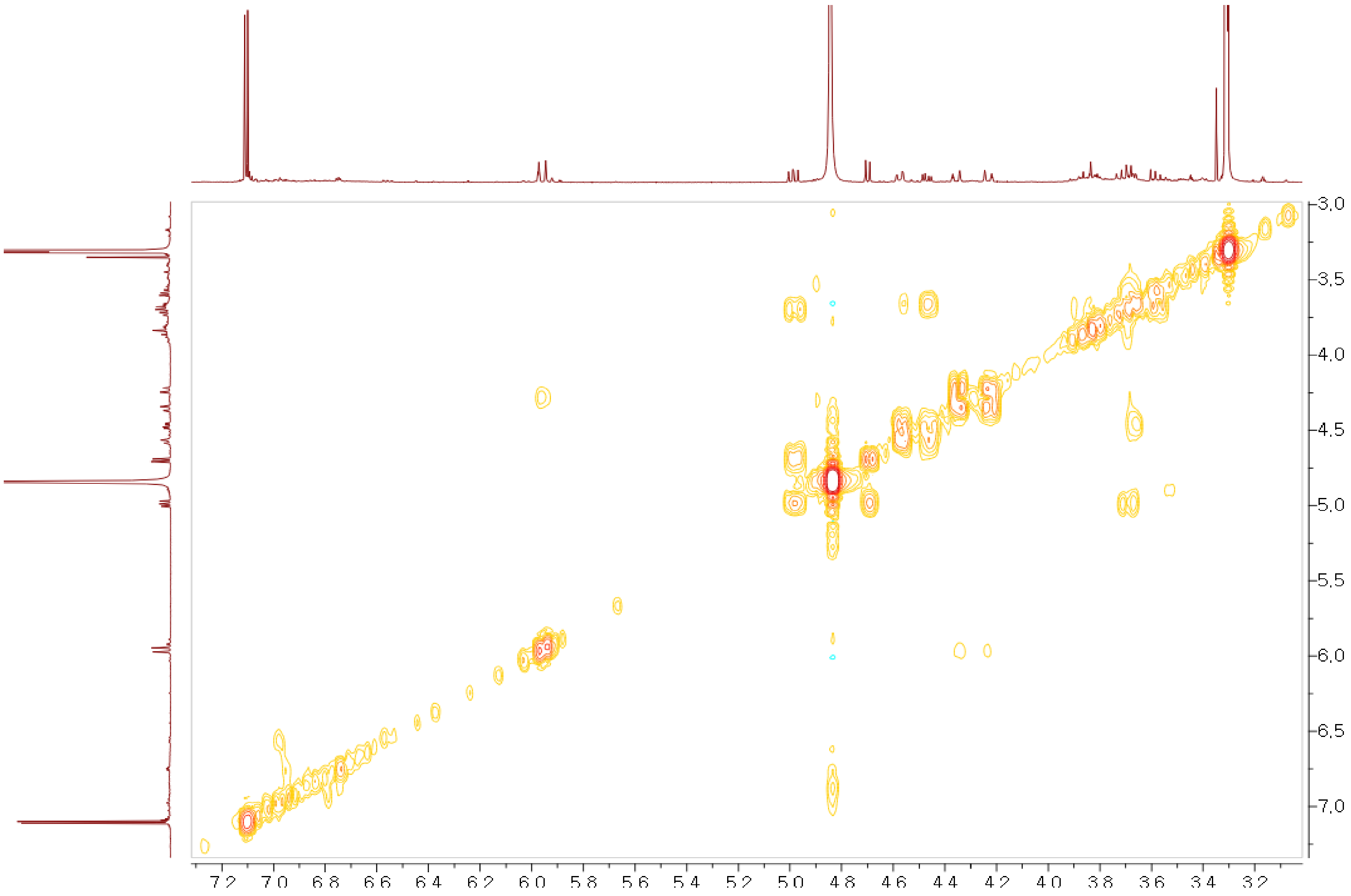

Figure S32 ^1H-^1H COSY data of **16** (CD_3OD)

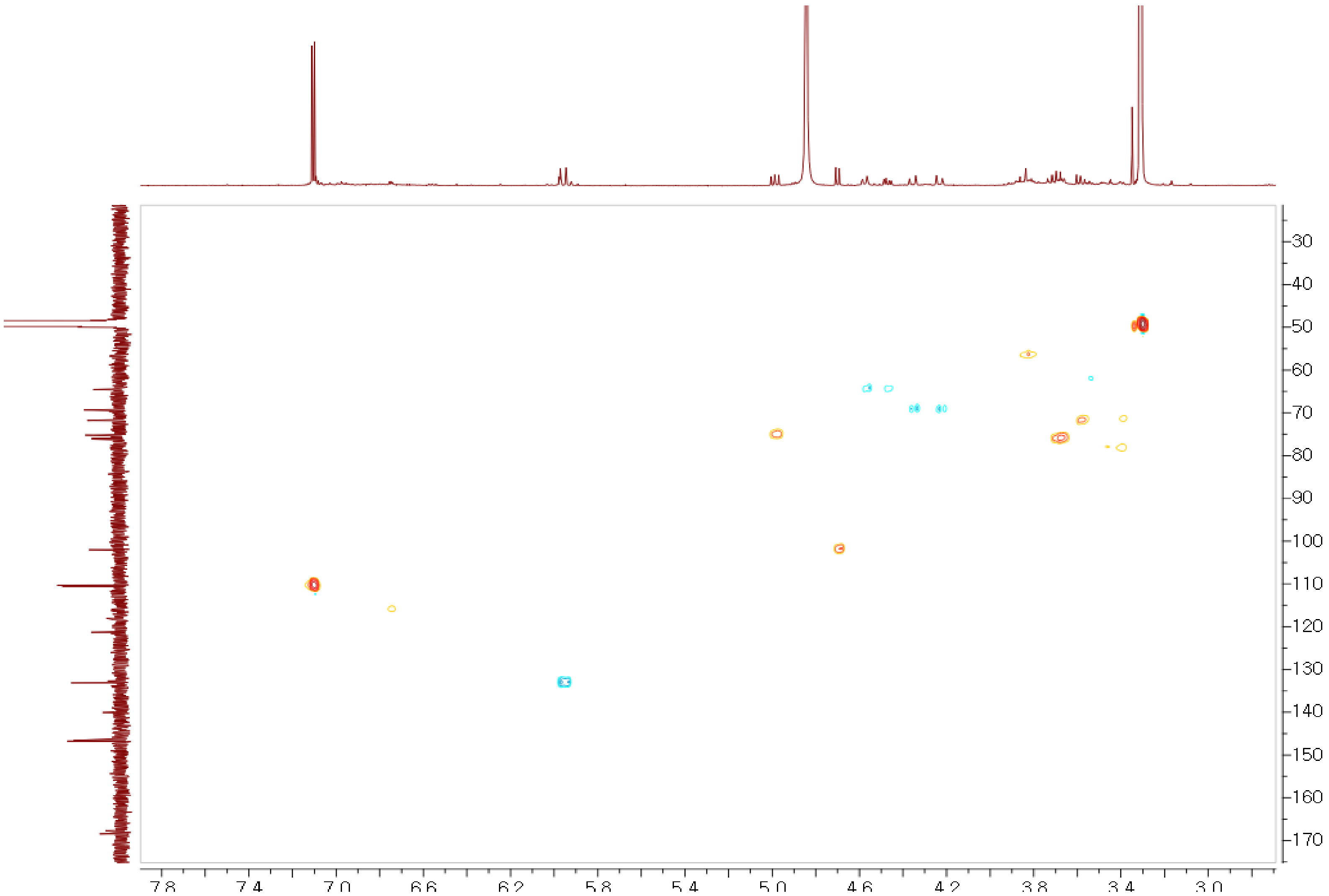

Figure S33 HSQC data of **16** (CD_3OD)

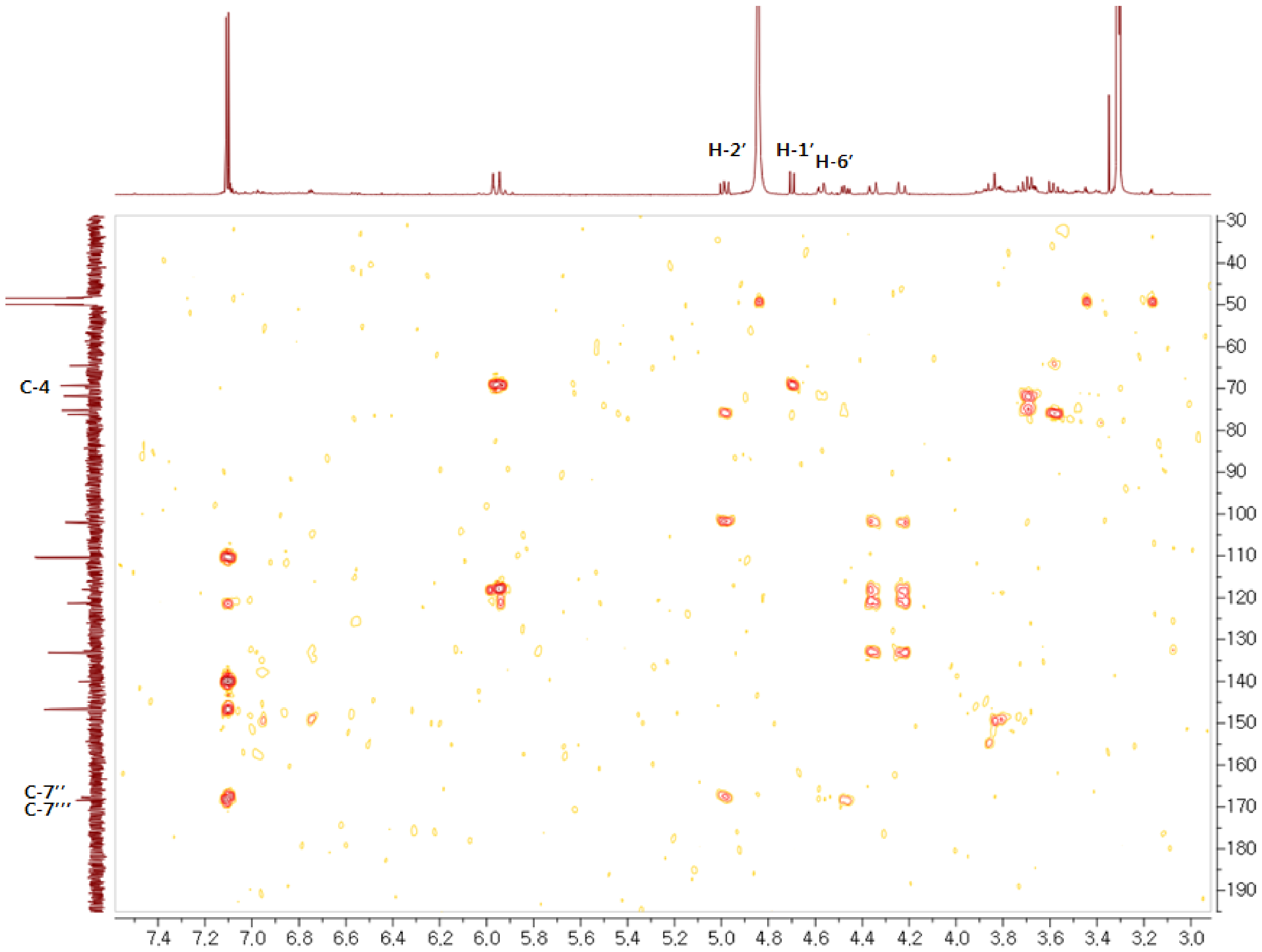

Figure S34 HMBC data of **16** (CD_3OD)
